# The perception of major life events across the life course

**DOI:** 10.1371/journal.pone.0314011

**Published:** 2024-12-04

**Authors:** Peter Haehner, Bernd Schaefer, Debora Brickau, Till Kaiser, Maike Luhmann

**Affiliations:** 1 Department of Psychology, Ruhr University Bochum, Bochum, Germany; 2 Department of Psychology, University of Zurich, Zürich, Switzerland; 3 Institute of Educational Science, University of Osnabrueck, Osnabrück, Germany; 4 German Center for Mental Health (DZPG), Bochum/Marburg, Germany; University of Bergamo, ITALY

## Abstract

To better understand the effects of life events, research interest recently turned to the question of how life events are perceived (e.g., as positive, predictable, or controllable). However, research on this topic primarily focused on young adulthood, leaving it unclear whether and how the perception of life events varies across the life course. In this study, we examined the relationship between age and different perceived event characteristics using nationally representative data from the German Socioeconomic Panel Innovation Sample (*N* = 1,044). We found that people reported different event types across among age groups. Furthermore, the perception of life events varied across age and depending on whether an event was experienced at a normative age or not. These findings underline the necessity to take on a life-course perspective when examining life events and support theoretical claims on the relevance of age norms in life-event research.

## Introduction

Major life events can trigger changes in various important life outcomes, including mental health, physical health, subjective well-being, and loneliness [1–3]. To better understand individual differences in these effects, research interest recently turned to the question of how major life events are perceived [e.g., 4–6]. However, to this date, research focused almost exclusively on young adulthood so that little is known regarding age differences in the perception of major life events [7–9]. In the present study, we take a step towards closing this research gap by examining whether and how the perception of major life events varies across the life course using data from the nationally representative German Socioeconomic Panel Innovation Sample (SOEP-IS).

### Major life events across the life course

Major life events such as starting a new relationship, a job loss, or the birth of a child are clearly timed, personally relevant experiences that disrupt one’s everyday life [5]. The effects of major life events have been examined in various disciplines including psychology, economics, epidemiology, and sociology [1, 10–12]. A theme cutting across research in these disciplines is that the effects of major life events depend on the timing of the event occurrence within people’s life course. Age-related differences in the occurrence and effects of major life events have been described and explained in theoretical accounts from various disciplines.

First, life script theory [13] posits that there are culturally shared expectations on the order and timing of life events. These life scripts describe an idealized life course based on social expectations on the most important events within different life phases [14–16]. Importantly, research on life scripts suggests that there are age-related differences in the valence of major life events. Specifically, positive life events like graduation or starting a romantic relationship are expected to occur primarily in young adulthood (i.e., positivity bump in young adulthood), whereas there are no clear age-graded expectations for negative events [17–19].

Second, the concept of developmental tasks has been applied to describe age-related differences in the occurrence and effects of life events [20, 21]. According to this perspective, major life events can be seen as markers of successful or unsuccessful fulfillment of such developmental tasks. For example, events such as job loss or starting a new employment can indicate a developmental milestone in establishing a career. Conceptualizing life events as the attainment of developmental tasks implies that there is a preferable timing of their occurrence throughout the life course and that the non-occurrence of events until a certain age may have adverse effects [21].

Third, and similarly, life course theory [22, 23] suggests the effects of major life events depend on when an event occurs within the life course. These age-graded differences in the effects of life events are assumed to depend on age norms, that is, social expectations regarding the timing of major life events [11, 22]. For example, graduating can be considered as normative in young adulthood but as non-normative in old adulthood. In line with life course theory, empirical research on age-normativity suggests that life events tend to have adverse effects when they occur at a non-normative age [24, 25]. Events that occur at a non-normative age are assumed to be characterized by little social guidance, reduced institutional support, or even social sanctions [24, 26, 27].

In summary, these theoretical perspectives outline the importance of considering age in life-event research as the effects of major life events differ depending on the timing of the event within people’s life course and age-graded social expectations. Thereby, the different theoretical accounts focus on the objective assessment of whether or not a major life event occurs at a certain age. However, recent research indicates that, to fully understand the effects of major life events, it is important to consider people’s subjective perception of major life events, which may also vary across age [e.g., 5, 6, 9].

### Age differences in the perception of major life events

Focusing on whether or not a major life event occurs provides a simple and objective approach to the assessment of major life events [e.g., 28–30]. However, this approach has been criticized as the nominally same event can be perceived quite differently by different individuals [5, 31]. For example, a relationship breakup might be a negative, unpredictable shock for one person but a positive relief for another. Different scholars thus have outlined the importance of assessing how people perceive major life events [e.g., 32–36]. Integrating existing approaches to the assessment of the perception of major life events, 5 [5] have developed a dimensional taxonomy of perceived event characteristics. This taxonomy comprises nine perceived event characteristics, which can be assessed reliably using the Event Characteristics Questionnaire (ECQ): *challenge*, *change in world views*, *emotional significance*, *external control*, *extraordinariness*, *impact*, *predictability*, *social status change*, and *valence*.

Recent longitudinal research has illustrated the relevance of these perceived event characteristics to understand the effects of major life events. For example, how people perceive major life events has been related to individual differences in changes in personality traits, subjective well-being, and depression [4, 6, 8, 9, 37, 38]. However, the lion’s share of this existing research examined the perception of major life events in young adulthood. Relatively little attention has been paid to age differences in the perception of major life events although there are reasons to assume that the perception of major life events may vary across the life course. Specifically, in line with the theoretical principles described above, age differences in the perception of major life events may arise due to age differences in the experienced event types and due to the normativity of events during certain life phases [11, 13, 22, 21]. Furthermore, there may be age differences in the perception style of life events. For example, personality traits and subjective well-being are assumed to influence how people perceive major life events [39]. As these constructs change across the life course [e.g., 40, 41], they might in turn contribute to age differences in how people perceive major life events. Thus, it seems warranted to examine whether and how the perception of major life events differs across age.

A better understanding of how the perception of major life events varies across age is important for several reasons. First, examining the perception of major life events across age is theoretically important. Different theoretical accounts posit that the effects of major life events differ across age due to age-graded differences in social expectations on the occurrence of events [13, 22]. Age differences in the perception of major life events could thus point to an important mechanism of this relationship. That is, social expectations may influence how people perceive major life events which, in turn, seems to predict the effects of major life events. Second, taking a life course perspective on the perception of major life events complements existing research on age differences in the occurrence and effects of major life events and connects research traditions from different fields of social sciences [42–47]. Third, evaluating age differences in the perception of major life events allows insights into the generalizability of existing findings on event-perception-outcome links [e.g., 4, 5]. If major life events are perceived differently across age (e.g., young adults generally perceive events as more challenging than old olds), one might expect that associations with individual differences in personality traits or subjective well-being also vary across age. Fourth, age differences in the perception of major life events could also be practically relevant. For example, if a major life event is perceived as particularly challenging or negative at a certain age, this finding might indicate that measures to prevent negative consequences of this event are particularly relevant at this age.

### The present study

In this preregistered study, we examined age differences in the perception of major life events using data from the SOEP-IS, a nationally representative and age-heterogeneous sample. In this dataset, participants reported the most important life event they had experienced in the last year and rated how they perceived this event. We addressed three research questions and tested five hypotheses.

First, do the reported event types differ across age groups (Research Question 1)? Replicating existing theoretical and empirical work on age differences in the occurrence of major life events [13, 22], we hypothesized that the frequency of the reported event types differs across age groups (Hypothesis 1).

Second, does the perception of major life events differ across age (Research Question 2)? Based on role script theory and findings on a positivity bump of events in young adulthood [13, 18], we hypothesized that the most important event of the last year is perceived more positively in young adulthood (Hypothesis 2a). Furthermore, based on theories and research indicating that personality traits, attitudes, and world-views become more stable over the life span [e.g., 40, 48–50], we hypothesized that life events are perceived as more world-view changing in young adulthood compared to middle adulthood or old adulthood (Hypothesis 2b).

Third, does the perception of specific event types differ across age and depending on whether an event is experienced at a normative age or not (Research Question 3)? For Research Question 3, we focused on the perception of specific event types (e.g., the death of a loved one) to examine whether and how the same type of event is perceived differently when it occurs in different life phases. As the occurrence of life events at a non-normative age runs against social norms and personal expectations [22, 24], we hypothesized that life events are perceived as less predictable, more extraordinary, and more challenging when they are experienced at a non-normative age (Hypotheses 3a to 3c).

## Materials and methods

### Transparency and openness

This study is based on the latest wave of the SOEP-IS (Year 2022). The dataset is licensed by the German Institute for Economic Research and may thus not be shared publicly. Researchers can access the dataset after signing a contract with the German Institute for Economic Research. The data is currently embargoed but will become available to the general public (expected in 2025). As the study used secondary data from the SOEP-IS ethical approval at Ruhr-University Bochum was not required (see [51] for details on ethical considerations).

The analyses for this manuscript were preregistered at https://osf.io/x3ucv. We had to deviate in one aspect from our preregistration: We had preregistered to examine age-specific effects (Research Question 3) for the five events with the highest sample size (death of a loved one, celebrating a special occasion, illness or injury, vacation, and childbirth). However, after revising our coding during the revision of this manuscript, the sample size for the event childbirth was too low (*N* = 17) to allow analyses for this event so that we decided to drop this event from our analysis of Research Question 3. Further details on the analyses (including R scripts) can be found at https://osf.io/qctxh. We report how we determined our sample size, all data exclusions (if any), all manipulations, and all measures in the study.

### Study design

The SOEP-IS is a longitudinal household survey administered by the German Institute for Economic Research. The SOEP-IS comprises a nationally representative sample of German inhabitants, and it is a shortened version of the longitudinal household panel study (SOEP-Core) with additional innovative modules. Data collection for the SOEP-IS takes place annually from September to December by visiting German households and interviewing all household members aged at least 16.

### Participants

Participants of the SOEP-IS were drawn using a random multistage procedure. In 2022, the total sample size of the SOEP-IS was *N* = 2,507. However, to reduce participant burden, only half of the participants received the innovative module on event perception. Furthermore, we excluded participants who did not provide a meaningful answer in the open-response field on the most important life event of the last year. Applying this exclusion criterion led to a final sample size of *N* = 1,044. The mean age of the sample was 56.24 years (*SD*_age_ = 17.68, *Min* = 18, *Max* = 95). 48% of participants were male and 52% were female.

### Measures

#### Event type

Using an open-response field, participants named the most important major life event they had experienced in the last 12 months. We categorized these free-text answers into event types. First, we created an initial coding scheme based on items of common life event checklist [e.g., 52] and coding procedures in other studies [4, 31]. Then, we tested and revised the coding scheme using 25% of the free-text responses. For example, we included new categories for event types that were not yet included and collapsed categories with a low frequency. Generally, we applied two different coding procedures: a fine-grained categorization (48 event types; e.g., *retirement*, *new job*) to provide detailed information on the experienced event types, and a broad categorization (12 event domains; e.g., *work*, *education*) to increase category-specific sample sizes and power for the analyses. Coding was done by two independent coders: The main coder coded 100% of the free-text answers. The reliability coder coded 33% of the answers, which were not included in the creation of the coding scheme. Interrater reliability was good (fine-grained categorization: κ = .85, broad categorization: κ = .76) so that we only used the codings of the main coder in the analyses. The coding instructions can be found in the OSF project of this article.

## Perceived event characteristics

The perception of the named event was assessed using the 9-item short version of the ECQ [5, 53]. Each perceived event characteristic was assessed with one item: *challenge* (“The event was stressful”), *emotional significance* (“The event was emotionally significant to me”), *external control* (“The event was in the hands of other people”), *extraordinariness* (“Most people like me experience this event sometime in their lives”, reverse-coded), *impact* (“The event had a strong impact on my life”), *predictability* (“The event occurred unexpectedly”, reverse-coded), *social status change* (“My reputation suffered from the event”), *valence* (“The event was negative”, reverse-coded), *change in world views* (“The event changed my world views”). Items were rated on a scale from 1 (*not true at all*) to 5 (*absolutely true*).

### Analyses

The analyses were conducted in R (Version 4.3.1). We used a level of significance of α = .05 for all analyses. For some analysis steps, we had to categorize age into age groups. As done by 21 [21], we distinguished three age groups: young adulthood (18–30 years, *N* = 105), middle adulthood (30–60 years, *N* = 456), and old adulthood (> 60 years, *N* = 483). However, to increase power and to examine the robustness of our findings, we repeated all analyses using terciles to create three age groups (young: 18–48.67 years, middle: 48.67–67, old: > 67 years).

### Research Question 1: Which events are reported as being most important?

To address Research Question 1, we calculated how often certain event types (fine-grained) or event domains (broad categorization) were mentioned in the different age groups. We used Fisher’s exact test to statistically examine the association between the categorical variables *event type/domain* and *age group* because expected frequencies were below 5 in more than 20% of cells. A significant test indicates that the frequencies of the reported event types/domains differ across age groups (cf. Hypothesis 1).

### Research Question 2: Does the perception of the most important major life event differ across age?

To address Research Question 2, we ran two different analyses. First, we explored the potentially nonlinear relationship between age and the perception of major life events using age as a metric predictor in *Generalized Additive Models* (GAMs). Second, we statistically compared the perception of major life events across different age groups to test our hypotheses (Hypotheses 2a and 2b).

*Age as a metric predictor*. GAMs can be used to easily fit and visualize (complex) nonlinear relationships between variables based on smoothing functions [54, 55]. These smoothing functions try to approximate non-linear trajectories in the data by balancing between over- and underfitting through a smoothing parameter. Using the R package *mgcv* [55], we estimated GAMs with a smoothed age term as predictor and with the nine perceived event characteristics as dependent variables. Among other things, GAMs provide information on (1) the *effective degrees of freedom* (*edf*) indicating the complexity of the relationship between age and a perceived event characteristic (i.e., higher values indicate that a higher-order polynomial regression would be needed to adequately reproduce the relationship), (2) the statistical significance of the relationship, and (3) *R*^*2*^ as an approximative indicator of the proportion of variance in a perceived event characteristic. We used *Restricted Maximum Likelihood*-method (REML) for model estimation. We did not limit the number of basic functions (*k*) in our analyses and relied on the default smoothing parameter of the *gam*-function. We explored possible problems in the estimation of GAMs using the *gam*.*check*-function. In a further step, we added event type as covariate (without smoothing). This model was used to explore the relationship between age and the perception of major life events controlling for the type of the reported event.

*Age groups*. As the results of the GAMs can be ambiguous regarding our hypotheses (e.g., there may be a complex nonlinear relationship between age and the perception of major life events), we additionally used one-way ANOVAs and post-hoc comparisons to test whether a perceived event characteristic differs across the three age groups (Hypotheses 2a and 2b).

### Research Question 3: Does the perception of specific event types differ across age and depending on whether an event is experienced at a normative age or not?

For Research Question 3, we conducted separate analyses for specific event types. To maximize power, we focused on those event types that had the highest sample sizes: vacation (*N* = 126), celebrating a special occasion (*N* = 84), illness or injury (*N* = 74), and death of a loved one (*N* = 71). For each of these events, we computed two analyses. First, we repeated the analyses described for Research Question 2 for these events to look at general age effects. Second, we examined age normativity effects using a modified age predictor in the GAMs. Specifically, we centered age on the average age at which the specific event was reported and used the absolute value of this centered variable (i.e., mean-age-deviation variable) as predictor in the GAMs. The mean ages of the four considered life events were as follows: vacation (*M* = 57.46 years, *SD* = 15.81), celebrating a special occasion (*M* = 59.59 years, *SD* = 17.93), illness or injury (*M* = 61.93 years, *SD* = 16.15), and death of a loved one (*M* = 59.34 years, *SD* = 14.87). GAMs using this mean-age-deviation variable as predictor were used to address the question of whether deviations from the mean age at which an event occurs are associated with the perception of major life events. We expected to find age normativity effects for the death of a loved one.

### Statistical power

Assuming a level of significance of α = .05 and a power to achieve of β = .80, we conducted power analyses to determine the minimum sample size to detect small (*R*^*2*^ = 1%), medium-sized (*R*^*2*^ = 4%), and strong effects (*R*^*2*^ = 9%) in our analyses [56]. For Research Question 1, power calculations suggested that the following sample sizes were needed to detect small, medium, and strong effects: *N*_*small*_ = 2,756, *N*_*medium*_ = 689, *N*_*large*_ = 307. Consequently, our analyses for Research Question 1 were sufficiently powered to detect medium-sized effects. For Research Questions 2 and 3, power calculations suggested that the following sample sizes were needed to detect small, medium, and strong effects: *N*_*small*_ = 1,094, *N*_*medium*_ = 275, *N*_*large*_ = 125. Consequently, our analyses for Research Question 2 were sufficiently powered to detect small effects, whereas our analyses for Research Question 3 could only detect (very) strong effects. The analyses for Research Question 3 should thus be regarded as preliminary because medium-sized or small effects could not be uncovered with sufficient power in these analyses.

## Results

**Fig 1:**
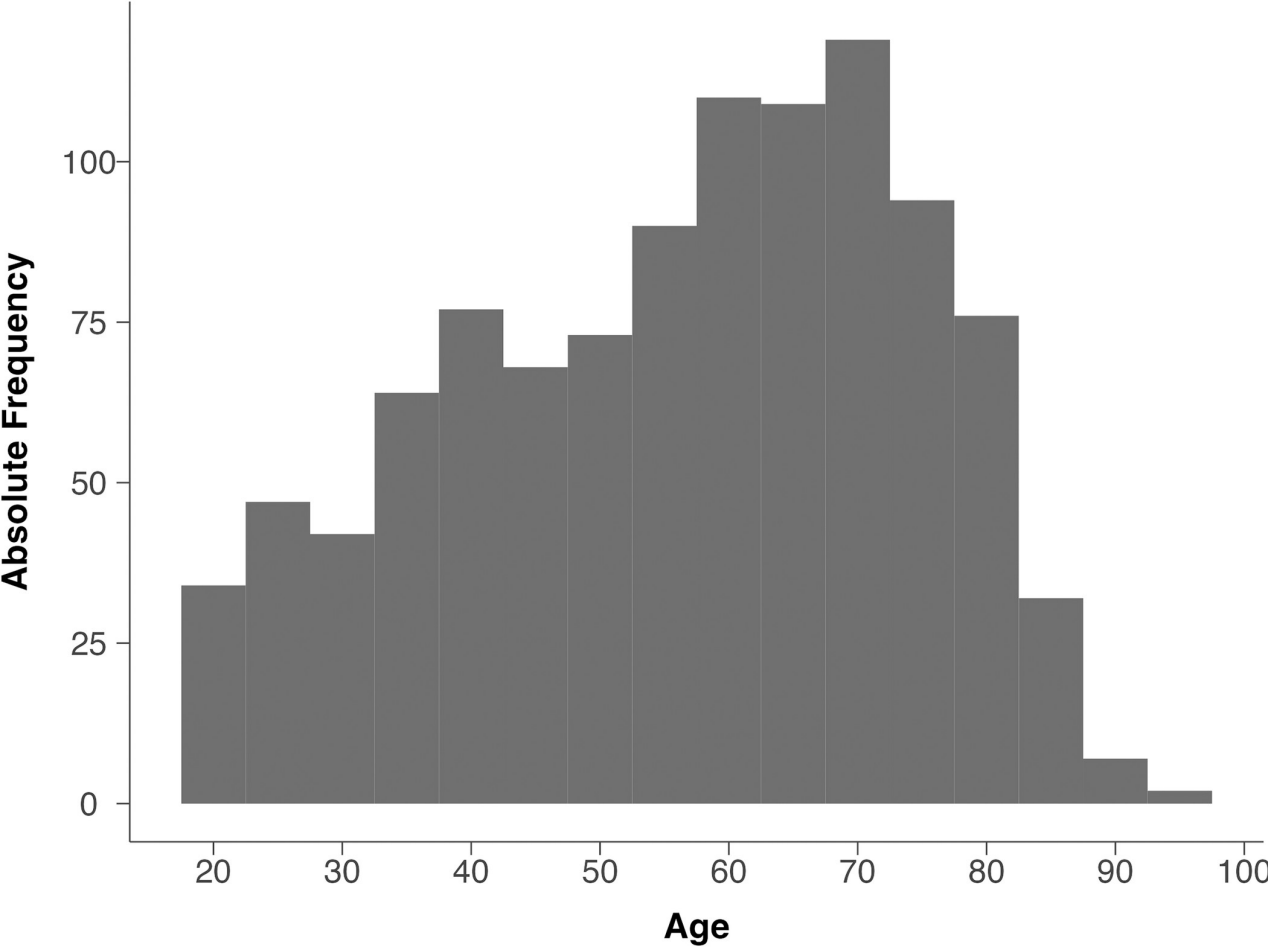
Histogram of participants’ age. This figure illustrates the age distribution in our representative dataset.

This article is accompanied by an HTML document (https://osf.io/qctxh/?view_only=01e12bbc2dab48d982afe85d0893a008) containing additional results such as descriptive statistics (Section 1). Fig 1 illustrates the age distribution in our sample.

### Research Question 1: Which events are reported as being most important?

**Fig 2:**
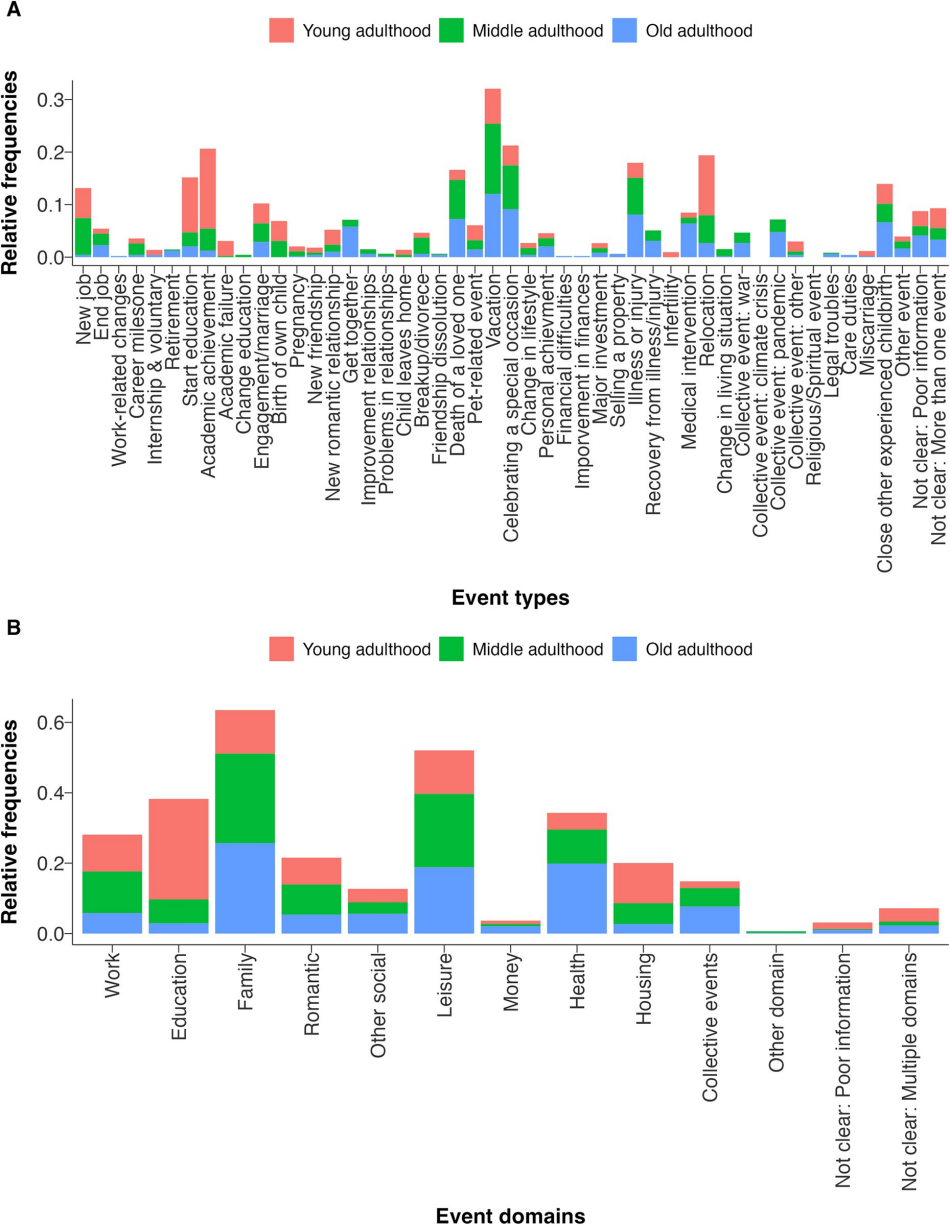
Research Question 1: Reported events in different age groups. This figure illustrates the relative frequencies of mentioned event types (Panel A) and event domains (Panel B) within the different age groups. That is, the bars indicate the proportion of events that were of a specific type or domain within an age group. This figure is based on the literature-based categorization of age (young adulthood: 18 to 30 years, middle adulthood: 30 to 60 years, old adulthood: > 60 years). Results for the terciles-based categorization of age and a colored version of this figure can be found in the S1 File (Section 2).

Consistent with Hypothesis 1, the reported event types differed significantly (*p*s < .001) across age groups (Fig 2). For example, in young adulthood, the events *starting education* or *academic achievement* were mentioned more frequently, whereas in old adulthood, the events *medical intervention* or *illness or injury* were mentioned more frequently. Similarly, there were significant differences in the mentioned event domains (*p*s < .001). For example, in young adulthood, more education-related events were reported, whereas in middle adulthood, work-related events were reported more frequently.

### Research Question 2: Does the perception of the most important major life event differ across age?

**Fig 3:**
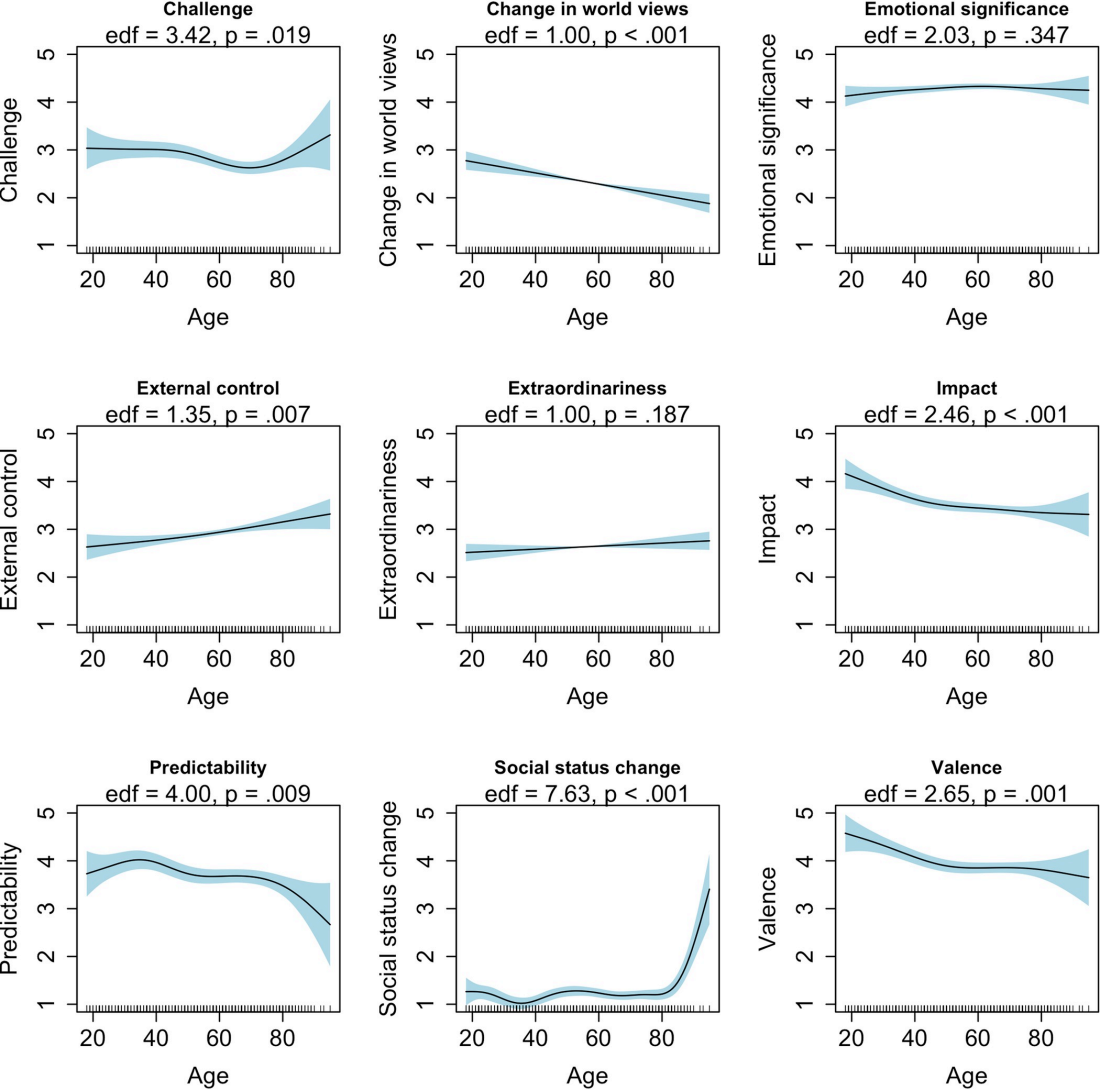
Research Question 2: The perception of the most important event across age. This figure illustrates how the perception of the most important event of the last year varies across age. Depicted are the results without event type as covariate. Results of models with event type as covariate can be found in Section 3 of the S1 File.

To address Research Question 2, we estimated GAMs using a smoothed age term as predictor and a perceived event characteristic as dependent variable. The results of these analyses are summarized in Table 1 and illustrated in Fig 3. Perceived challenge (*edf* = 3.42, *p* = .019), impact (*edf* = 2.64, *p* < .001), predictability (*edf* = 4.00, *p* = .009), social status change (*edf* = 7.63, *p* < .001), and valence (*edf* = 2.65, *p* = .001) varied in a non-linear fashion across age (see Fig 3 for details regarding the shape of the association). For example, the perceived (positive) valence of the most important event decreased until age 50, was stable between age 50 to 80, and decreased again from age 80 onwards. Furthermore, change in world views decreased (*edf* = 1.00, *p* < .001) and external control increased (*edf* = 1.35, *p* = .007) in a linear fashion across age, whereas extraordinariness (*edf* = 1.00, *p* = .187) and emotional significance (*edf* = 2.03, *p* = .347) did not vary significantly across age. In general, age explained a small to medium-sized proportion of the variance of the perceived event characteristics (.00 ≤ *R*^*2*^ ≤ .04; 56).

**Table 1:** Results of research Question 2: Age differences in perception of most important event.

Perceived event characteristic	Without covariate			Controlled for event type		
	*Edf*	*p*	*R* ^*2*^	*Edf*	*p*	*R* ^*2*^
Challenge	**3.42**	**.019**	.011	**2.43**	**.007**	.326
Change in world views	**1.00**	**< .001**	.020	**1.00**	**< .001**	.170
Emotional significance	2.03	.347	.002	2.12	.463	.085
External control	**1.35**	**.007**	.009	**1.00**	**.008**	.162
Extraordinariness	1.00	.187	.001	1.00	.201	.100
Impact	**2.46**	**.000**	.021	**2.36**	**.001**	.145
Predictability	**4.00**	**.009**	.014	1.01	.713	.362
Social status change	**7.63**	**.000**	.044	**6.42**	**.011**	.151
Valence	**2.65**	**.001**	.016	2.06	.276	.565

*Note*. This table summarizes the results of GAMs using a smoothed age term as predictor and a perceived event characteristics as dependent variable. *Edf* (effective degrees of freedom) are an indicator of the complexity of the relationship between age and a perceived event characteristics (i.e., higher values indicate that a higher-order polynomial regression would be needed to adequately reproduce the relationship). The statistical significance of the smooth term indicates whether age is related to a perceived event characteristic. Significant effects (α = .05) are indicated in bold. Finally, *R*^*2*^ is an approximative indicator of the proportion of variance that is explained by the included predictors.

To test our hypotheses, we also used a categorical classification of age (see Section 3 of the S1 File). In line with Hypothesis 2a, we found that the most important event of the last year was perceived as significantly more positive in young adulthood compared to middle adulthood (*b* = 0.58, *p*_*Tukey*_ = .003) or old adulthood (*b* = 0.62, *p*_*Tukey*_ = .001). Furthermore, in line with Hypothesis 2b, we found that the most important event of the last year was perceived as significantly more world-view changing in young adulthood compared to old adulthood (*b* = 0.64, *p*_*Tukey*_ < .001). However, contrary to our hypothesis, the comparison between young adulthood and middle adulthood did not reach statistical significance (*b* = 0.32, *p*_*Tukey*_ = .094).

In the next step, we additionally included event type as covariate in the models to examine whether age differences in the perception of major life events can be found after accounting for the fact that the experienced events differ across age. As can be seen in Table 1, models including event type as covariate generally provided similar results as models not accounting for event type. There were only two exceptions: After controlling for event type, perceived predictability and valence of the most important event no longer varied across age. Thus, results indicate that age differences in perceived predictability and valence may be explained by age differences in the reported event types, whereas age differences in other perceived event characteristics do not (only) depend on the reported event type. Of note, models including event types as covariate explained a substantially larger amount of variance (.08 ≤ *R*^*2*^ ≤ .57), indicating that event type may be a better indicator of the perception of major life events than age alone.

### Research Question 3: Does the perception of specific event types differ across age and depending on whether an event is experienced at a normative age or not?

**Fig 4:**
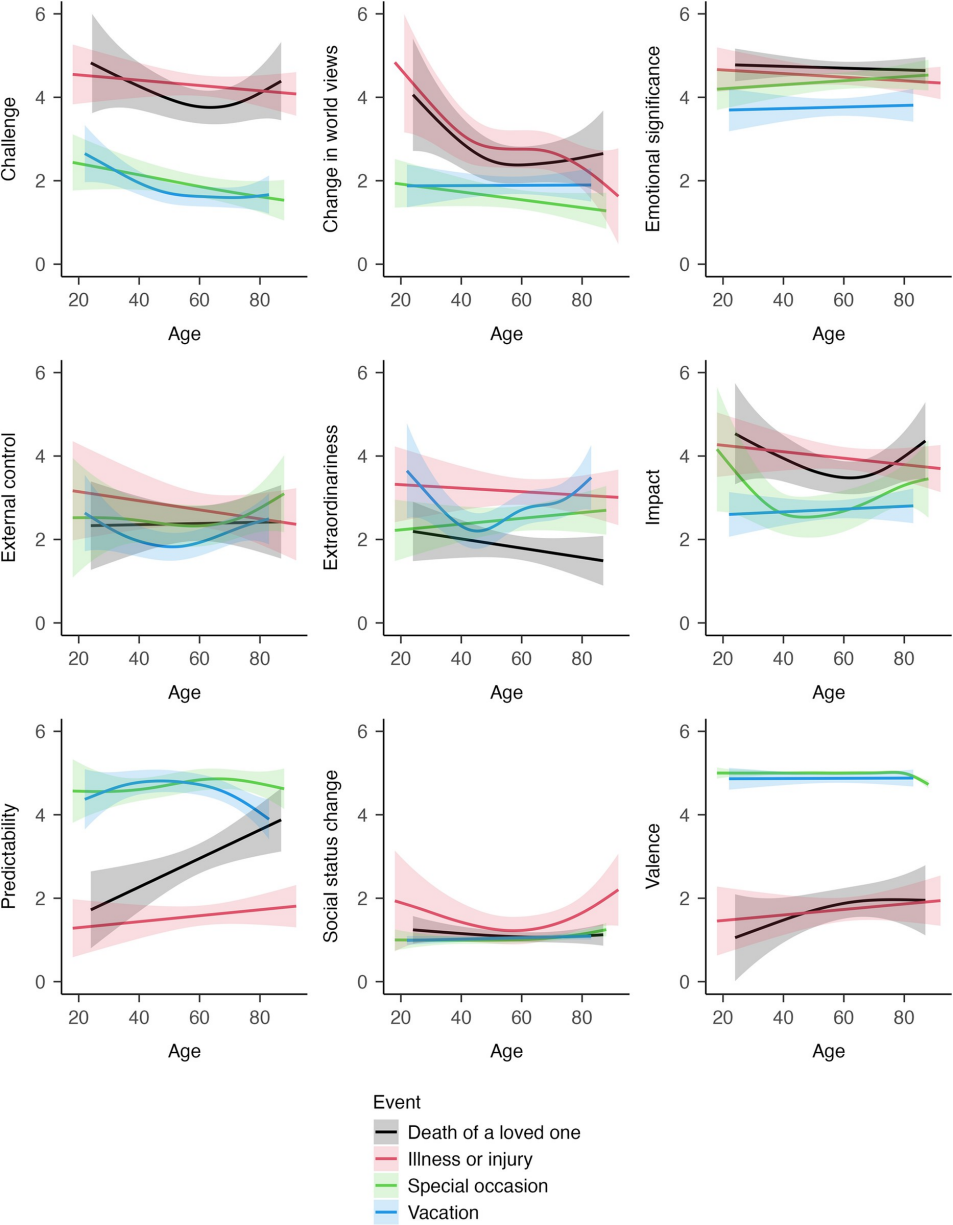
Research Question 3a: Age differences in the perception of specific events. This figure illustrates how the perception of specific event types varies across age. For a colored version of this figure, please see Section 4 of the S1 File.

To address Research Question 3, we focused on the event types with the largest sample sizes (vacation, celebrating a special occasion, illness or injury, death of a loved one). First, we examined whether the perception of specific events varies across age. Results are summarized in Table 2 and illustrated in Fig 4. The overall finding is that age differences in the perception of major life events differed among event types. For example, while perceived extraordinariness of a vacation decreased during young adulthood, reached a nadir in middle adulthood, and then increased again throughout the remainder of the lifespan, no statistically significant age differences were found for the perceived extraordinariness of illness/injury, celebrations, or death of a loved one. These results indicate that although there may be general age trends in the perception of the most important event (Research Question 2), there nonetheless seem to be specific patterns for specific major life events.

**Table 2:** Research Question 3a: Age differences in the perception of specific major life events.

Perceived event characteristic	Vacation			Celebrating a special occasion			Illness or injury			Death of a loved one		
	*Edf*	*p*	*R* ^*2*^	*Edf*	*p*	*R* ^*2*^	*Edf*	*p*	*R* ^*2*^	*Edf*	*p*	*R* ^*2*^
Challenge	**2.25**	**.033**	.061	1.12	.079	.032	1.00	.420	-.005	2.12	.250	.040
Change in world views	1.00	.962	-.008	1.00	.154	.012	**2.71**	**.026**	.119	2.22	.148	.060
Emotional significance	1.00	.781	-.007	1.00	.391	-.003	1.00	.457	-.006	1.00	.681	-.012
External control	2.35	.163	.034	1.95	.554	.013	1.00	.406	-.004	1.00	.919	-.015
Extraordinariness	**3.40**	**.038**	.084	1.00	.424	-.005	1.00	.672	-.011	1.00	.245	.006
Impact	1.00	.633	-.006	2.75	.121	.067	1.00	.362	-.002	2.29	.184	.054
Predictability	**2.52**	**.023**	.072	2.14	.490	.019	1.00	.350	-.002	**1.00**	**.007**	.089
Social status change	1.00	.234	.003	2.27	.076	.070	2.29	.217	.048	1.57	.572	.003
Valence	1.00	.939	-.008	**6.08**	**.001**	.255	1.00	.466	-.006	1.52	.307	.021

*Note*. This table summarizes the results of GAMs using a smoothed age term as predictor and a perceived event characteristics as dependent variable for specific major life events. *Edf* (effective degrees of freedom) are an indicator of the complexity of the relationship between age and a perceived event characteristics (i.e., higher values indicate that a higher-order polynomial regression would be needed to adequately reproduce the relationship). The statistical significance of the smooth term indicates whether age is related to a perceived event characteristic. Significant effects (α = .05) are indicated in bold. Finally, *R*^*2*^ is an approximative indicator of the proportion of variance that is explained by the included predictors. *R*^*2*^ of GAMs can be negative if a model is worse than a one parameter constant model.

^a^ GAMs using perceived social status changes as dependent variable did not converge for this event.

**Fig 5:**
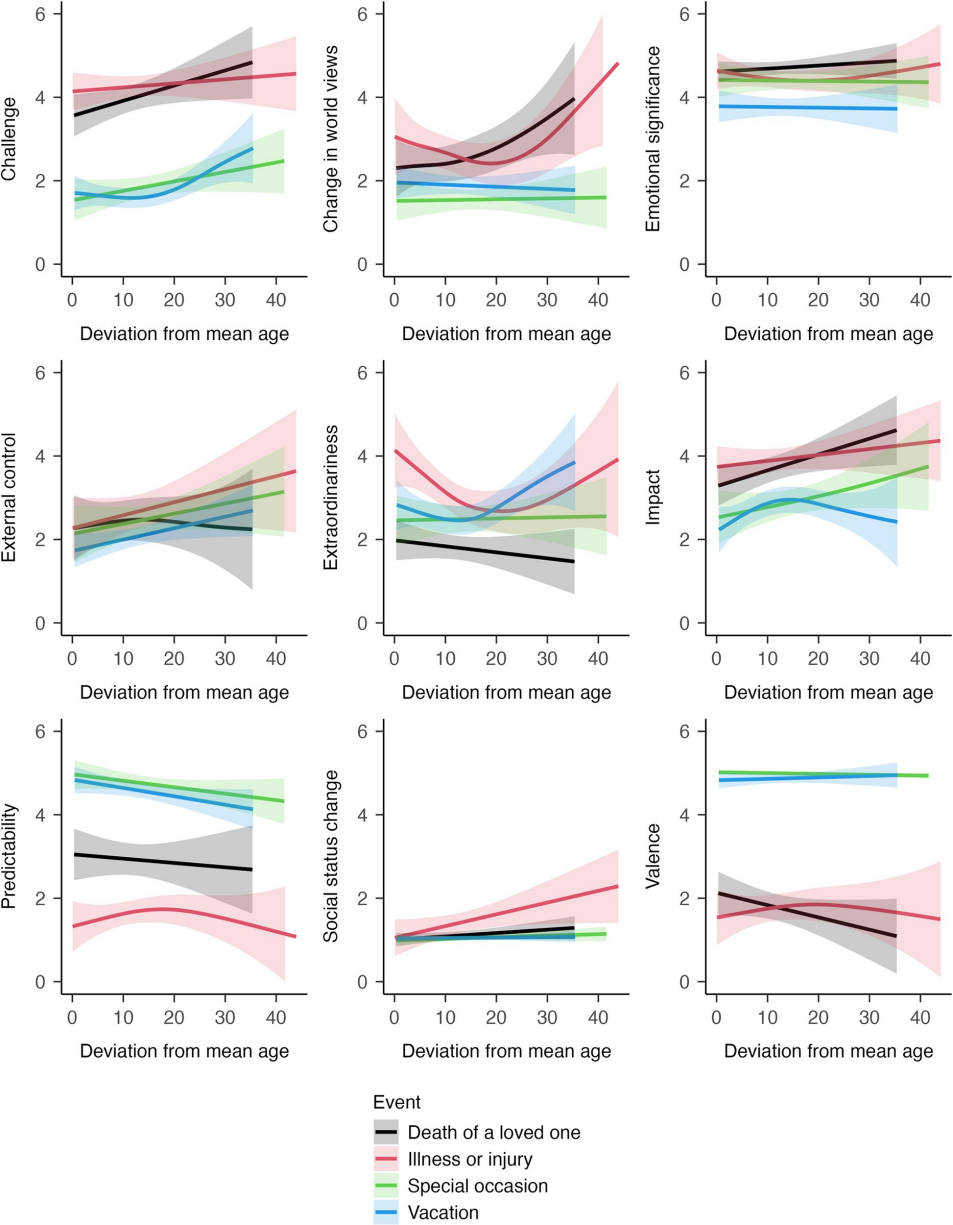
Research Question 3b: Differences in the perception of major life events depending on age-normativity. This figure illustrates how the perception of specific event types varies depending on whether the event is experienced at a normative age or not. For a colored version of this figure, please see Section 4 of the S1 File.

Second, we examined whether the perception of specific events depends on whether an event is experienced at a normative age or not. Results are summarized in Table 3 and illustrated in Fig 5. We had hypothesized that the death of a loved one is perceived as more challenging, more extraordinary, and less predictable when it is experienced at a non-normative age (Hypotheses 3a to 3c). Results were only partly in line with these hypotheses. The death of a loved one was experienced as more challenging when experienced at a non-normative age (*edf* = 1.00, *p* = .043). However, the other hypothesized associations were not found. Generally, the results of Research Question 3 should be regarded as preliminary due to the relatively small sample sizes for specific event types.

**Table 3:** Research Question 3b: Differences in the perception of major life events depending on age normativity.

Perceived event characteristic	Vacation			Celebrating a special occasion			Illness or injury			Death of a loved one		
	*Edf*	*p*	*R* ^*2*^	*Edf*	*p*	*R* ^*2*^	*Edf*	*p*	*R* ^*2*^	*Edf*	*p*	*R* ^*2*^
Challenge	**2.31**	**.034**	.060	1.00	.118	.017	1.00	.506	-.008	**1.00**	**.043**	.045
Change in world views	1.00	.685	-.007	1.00	.885	-.012	2.50	.137	.065	1.69	.093	.058
Emotional significance	1.00	.892	-.008	1.00	.912	-.012	1.71	.538	.005	1.00	.398	-.004
External control	1.00	.040	.026	1.00	.230	.006	1.00	.192	.010	1.37	.875	-.008
Extraordinariness	2.38	.075	.051	1.00	.892	-.013	**2.46**	**.043**	.099	1.00	.375	-.003
Impact	2.32	.176	.032	1.15	.154	.019	1.00	.362	-.002	**1.00**	**.028**	.056
Predictability	1.01	.056	.022	1.00	.127	.016	1.88	.447	.014	1.00	.632	-.011
Social status change	1.00	.703	-.007	1.00	.266	.003	**1.00**	**.048**	.041	1.00	.157	.015
Valence	1.00	.592	-.006	1.00	.138	.014	1.53	.651	-.001	1.00	.110	.023

*Note*. This table summarizes the results of GAMs using the deviation from the mean age of event occurrence as predictor and a perceived event characteristics as dependent variable for specific major life events. *Edf* (effective degrees of freedom) are an indicator of the complexity of the relationship between this mean-age-deviation variable and a perceived event characteristics (i.e., higher values indicate that a higher-order polynomial regression would be needed to adequately reproduce the relationship). The statistical significance of the smooth term indicates whether the mean-age-deviation variable is related to a perceived event characteristic. Significant effects (α = .05) are indicated in bold. Finally, *R*^*2*^ is an approximative indicator of the proportion of variance that is explained by the included predictors. *R*^*2*^ of GAMs can be negative if a model is worse than a one parameter constant model.

## Discussion

Based on a nationally representative and age-heterogeneous German sample, we examined how major life events are perceived over the life course. In the following, we summarize the most important results of our study and discuss possible explanations and potential implications of these findings.

First, we found that the reported event types and event domains differed across age groups. In line with theoretical predictions [13, 22, 21], these age differences in the reported events match social expectations. For example, health-related events were reported more frequently in old adulthood whereas education-related events were reported more frequently in young adulthood. These findings underline the relevance of considering age when examining the effects of major life events, and they support theoretical notions on age differences in the timing of life events throughout people’s life course [13, 22].

Second, there were age differences in the perception of the most important event, and these differences were not solely attributable to differences in the experienced event types. Specifically, we found that the reported events were perceived more positively in young adulthood compared to old adulthood. This result is in line with life script theory, which suggests that young adulthood is a life phase that is characterized by many positive life transitions [e.g., 13, 14, 17]. Importantly, this positivity bump in young adulthood is assumed to be practically relevant as it seems to be related to personality changes into the direction of greater maturity in young adulthood [21, 40, 50]. However, memory bias or reporting bias could be alternative explanations for this finding. For example, drawing on mood-congruent memory effect, it might be the case that young adults report more positive events than old adults as they can retrieve them more easily due to the on average more positive mood in young adulthood [41, 57]. Furthermore, we found that the reported major life events were perceived as more world-view changing in young adulthood compared to old adulthood. These findings match research on the development of attitudes, traits, and narratives [e.g., 40, 49]. As these constructs become more stable when people age, major life events might have a reduced impact on people’s world views and, therefore, be perceived as less world-view changing. More generally, the finding that several perceived event characteristics varied across age even when controlling for event type could be explained by the fact that variables associated with (or possibly *causing*) the perception of major life events change across the lifespan. For example, personality traits, subjective well-being, or cognitive styles have been assumed to predict the perception of major life events [8, 9, 58]. As these variables change across the lifespan, they might lead to age differences in the perception style of major life events.

Third, we found that the perception of major life events also partly differed depending on whether an event was experienced at a normative age or not. For example, the death of a loved one was perceived as more challenging when experienced at a non-normative age. These findings support theoretical notions on the relevance of age norms when examining the effects of major life events [22, 24, 26, 44]. The perception of major life events could be a mechanism linking age norms to detrimental outcomes: Experiencing life events at a non-normative age might lead to a more unfavourable event perception, which in turn may cause more maladaptive reactions to the event [9, 39]. However, it should be noted that age-normativity differences in the perception of major life events were relatively small and that power for these analyses was limited. Furthermore, we only examined “statistical norms” in this study, that is, we defined age normativity based on the mean age of event occurrence, but we did not consider actual prescriptions related to age (e.g., an event *should* happen until a certain age; [11].

In summary, our findings suggest that age differences in the perception of major life events depend on the experienced event types, differences in the perception style of the same event, and age-differences related to the normativity of life events in certain life phases. Thus, age seems to be a relevant variable not only regarding the occurrence of major life events but also when examining the perception of major life events [21, 22, 44]. Consequently, research on how the perception of major life events is related to changes in important life outcomes such as health, relationship quality, or well-being should move beyond examining these links in young adulthood and instead investigate event-perception-outcome links across the entire lifespan. The findings of the present study are also practically relevant as they provide insights into who might be at risk for unfavorable event perceptions. Specifically, people above age 80 tend to perceive life events as more challenging, more social status threatening, and more negative, indicating that adaptation processes to life events may take place in older adulthood.

### Strengths and limitations

The present study used a nationally representative dataset to examine the relationship between age and the perception of major life events. Using GAMs, we accounted for the possible nonlinear relationship between these constructs and considered both age effects and age-normativity effects. However, the present study also had several limitations. First, participants were asked to report the most important event that they had experienced in the last year. Thus, it is unclear whether our findings generalize to other experienced life events with a lower importance. Relatedly, the results of the present study may be influenced by the historical context. The data was collected in 2022 and several participants named events related to the Covid-19 pandemic or the Russian-Ukrainian war. As these collective life events affected people of any age, we might have underestimated age differences in the present study.

Second, although we relied on a relatively large, nationally representative study, the sample sizes of specific event types were still quite low, which limited our power to uncover age differences and age-normativity effects for specific life events. Thus, results regarding Research Question 3 should be regarded as preliminary.

Third, in the SOEP-IS, each perceived event characteristic was only assessed with one item. While there is initial evidence supporting the psychometric quality of this brief measure of perceived event characteristics [53], longer measures are required for a broader coverage of a construct and to obtain more reliable estimates. Specifically, the item used to assess extraordinariness (“Most people like me experience this event sometime in their lives”, reverse-coded) may not be ideal to identify age-normativity effects. Findings related to extraordinariness may be seen as “the lower bound” of effects that can be found for this event characteristic.

Fourth, this study relied on a sample recruited in a Western, democratic country. However, the normativity of life events differs across cultures [e.g., 59]. Consequently, research on the perception of major life events in other cultural contexts is needed to test whether the results of the present study generalize to other cultural contexts.

## Conclusion

The perception of major life events varies across the life course and partly also depending on whether an event is experienced as a normative age or not. The perception of major life events could thus be a relevant mechanism linking age norms to age-graded differences in the effects of major life events. Therefore, future research on the perception of major life events should broaden its scope beyond young adulthood and examine event-perception-outcome links across the entire lifespan.

## Supporting information

S1 File This manuscript is accompanied by an HTML document with supporting information containing our analysis code, additional figures, and tables. (HTML)
