# Supplementary material for: The perception of major life events across the life course
Source: PLoS One. 2024 Dec 4;19(12):e0314011. doi: 10.1371/journal.pone.0314011 (PMC11616814; doi:10.1371/journal.pone.0314011)
Supplement: S1 File — (HTML) [file pone.0314011.s001.html]

Results


Code 

- Show All Code
- Hide All Code

# Results

### Age Differences in the Perception of Major Life Events

####

# Section 1: Descriptive Statistics

## Experienced event types

```
freq_type <- as.data.frame(table(data$event_type))
names(freq_type) <- c("Event type", "N")
  
kable(freq_type
      , caption="Absolute frequencies of experienced event types"
      , escape=FALSE
      , label = NA) %>% 
  kable_styling(bootstrap_options = c("striped", "hover"),  
                fixed_thead = T, full_width = TRUE, position="left")
```

Absolute frequencies of experienced event types

| Event type | N |
| --- | --- |
| New job | 40 |
| End job | 22 |
| Work-related changes | 1 |
| Career milesone | 13 |
| Internship & voluntary | 3 |
| Retirement | 7 |
| Start education | 33 |
| Academic achievement | 41 |
| Academic failure | 4 |
| Change education | 2 |
| Engagement/marriage | 34 |
| Birth of own child | 18 |
| Pregnancy | 6 |
| New friendship | 5 |
| New romantic relationship | 14 |
| Get together | 34 |
| Improvement relationships | 7 |
| Problems in relationships | 3 |
| Child leaves home | 3 |
| Breakup/divorece | 18 |
| Friendship dissolution | 3 |
| Death of a loved one | 71 |
| Pet-related event | 18 |
| Vacation | 126 |
| Celebrating a special occasion | 86 |
| Change in lifestyle | 9 |
| Personal achievment | 18 |
| Financial difficulties | 1 |
| Imporvement in finances | 1 |
| Major investment | 9 |
| Selling a property | 3 |
| Illness or injury | 74 |
| Recovery from illness/injury | 24 |
| Infertility | 1 |
| Medical intervention | 37 |
| Relocation | 49 |
| Change in living situation | 7 |
| Collective event: war | 22 |
| Collective event: climate crisis | 0 |
| Collective event: pandemic | 34 |
| Collective event: other | 7 |
| Religious/Spiritual event | 0 |
| Legal troubles | 4 |
| Care duties | 2 |
| Miscarriage | 2 |
| Close other experienced childbirth | 52 |
| Other event | 15 |
| Not clear: Poor information | 31 |
| Not clear: More than one event | 30 |

## Experienced event domains

```
freq_domain <- as.data.frame(table(data$event_domain))
names(freq_domain) <- c("Event domain", "N")
  
kable(freq_domain
      , caption="Absolute frequencies of experienced event domains"
      , escape=FALSE
      , label = NA) %>% 
  kable_styling(bootstrap_options = c("striped", "hover"),  
                fixed_thead = T, full_width = TRUE, position="left")
```

Absolute frequencies of experienced event domains

| Event domain | N |
| --- | --- |
| Work | 93 |
| Education | 75 |
| Family | 253 |
| Romantic | 73 |
| Other social | 46 |
| Leisure | 199 |
| Money | 14 |
| Health | 145 |
| Housing | 52 |
| Collective events | 63 |
| Other domain | 3 |
| Not clear: Poor information | 8 |
| Not clear: Multiple domains | 20 |

## Perceived event characteristics

```
desc_pec <- round(psych::describe(data.frame(data$ecq.chall, data$ecq.changeww,
                                       data$ecq.emosig, data$ecq.extcon,
                                       data$ecq.extrao, data$ecq.impact,
                                       data$ecq.predict, data$ecq.socstat,
                                       data$ecq.val))[, 2:4],2)
names(desc_pec) <- c("N", "M", "SD")
row.names(desc_pec) <- c("Challenge", "Change in world views", 
                        "Emotional significance", "External control", 
                        "Extraordinariness", "Impact",
                        "Predictability", "Social status change", 
                        "Valence")

kable(desc_pec
      , caption="Descriptive statistics perceived event characteristics"
      , escape=FALSE
      , label = NA) %>% 
  kable_styling(bootstrap_options = c("striped", "hover"), 
                fixed_thead = T, full_width = FALSE, position="left")
```

Descriptive statistics perceived event characteristics

|  | N | M | SD |
| --- | --- | --- | --- |
| Challenge | 1039 | 2.83 | 1.59 |
| Change in world views | 1039 | 2.33 | 1.44 |
| Emotional significance | 1042 | 4.28 | 1.03 |
| External control | 1033 | 2.92 | 1.64 |
| Extraordinariness | 996 | 2.63 | 1.34 |
| Impact | 1040 | 3.52 | 1.36 |
| Predictability | 1036 | 3.72 | 1.60 |
| Social status change | 1037 | 1.21 | 0.71 |
| Valence | 1038 | 3.96 | 1.62 |

## Age

```
ggplot(data = data, aes(x = age)) +
  geom_histogram(binwidth = 5) +
  scale_x_continuous(breaks = c(20, 30, 40, 50, 60, 70, 80, 90, 100)) +
  theme_pub() +
  labs(x = "Age", y = "Absolute Frequency")
```

# Section 2: Research Question 1 - Reported events in different age groups

## Literature-based classification of age

### Event domains

#### Table and statistical test

```
t1 <- table(data$event_domain, data$age.theory)

kable(t1
      , caption="Frequencies of event domains by age group"
      , escape=FALSE
      , label = NA) %>% 
  kable_styling(bootstrap_options = c("striped", "hover", "condensed"), 
                fixed_thead = T, full_width = FALSE, position="left")
```

Frequencies of event domains by age group

|  | Young adulthood | Middle adulthood | Old adulthood |
| --- | --- | --- | --- |
| Work | 11 | 54 | 28 |
| Education | 30 | 31 | 14 |
| Family | 13 | 116 | 124 |
| Romantic | 8 | 39 | 26 |
| Other social | 4 | 15 | 27 |
| Leisure | 13 | 95 | 91 |
| Money | 1 | 3 | 10 |
| Health | 5 | 44 | 96 |
| Housing | 12 | 27 | 13 |
| Collective events | 2 | 24 | 37 |
| Other domain | 0 | 2 | 1 |
| Not clear: Poor information | 2 | 1 | 5 |
| Not clear: Multiple domains | 4 | 5 | 11 |

```
## Check assumption
ratio.low.freq <- sum(chisq.test(table(data$event_domain, data$age.theory))$expected <= 5)/length(chisq.test(table(data$event_domain, data$age.theory))$expected)
# Fishers exact test recommended

## Results
fisher.test(table(data$event_domain, data$age.theory), simulate.p.value = TRUE)
```

```
## 
##  Fisher's Exact Test for Count Data with simulated p-value (based on
##  2000 replicates)
## 
## data:  table(data$event_domain, data$age.theory)
## p-value = 0.0004998
## alternative hypothesis: two.sided
```

The mentioned event domains differ significantly between age groups.

#### Graphs

Absolute frequencies:

```
ggplot(aes(x = event_domain), data = data) +
  geom_bar(aes(fill = data$age.theory)) +
  labs(y = "Frequencies", x = "Event domains") +
  theme_pub() +
  theme(axis.text.x = element_text(angle = 90, vjust = 0.5, hjust=1)) +
  theme(legend.text=element_text(size=13))
```

Relative frequencies:

```
prop <- as.data.frame(prop.table(table(data$event_domain, data$age.theory), margin = 2))

p1 <- ggplot(aes(x = Var1, y = Freq), data = prop) +
  geom_col(stat = identity, aes(fill = Var2)) +
  labs(y = "Relative frequencies", x = "Event domains") +
  theme_pub() +
  theme(axis.text.x = element_text(angle = 90, vjust = 0.5, hjust=1)) +
  theme(legend.text=element_text(size=13))

p1
```

#### Residuals

```
t2 <- round(chisq.test(table(data$event_domain, data$age.theory))$residuals,2)

kable(t2
      , caption="Residuals of event domains by age group"
      , escape=FALSE
      , label = NA) %>% 
  kable_styling(bootstrap_options = c("striped", "hover", "condensed"), 
                fixed_thead = T, full_width = FALSE, position="left")
```

Residuals of event domains by age group

|  | Young adulthood | Middle adulthood | Old adulthood |
| --- | --- | --- | --- |
| Work | 0.54 | 2.10 | -2.29 |
| Education | 8.18 | -0.31 | -3.51 |
| Family | -2.47 | 0.52 | 0.64 |
| Romantic | 0.24 | 1.26 | -1.34 |
| Other social | -0.29 | -1.14 | 1.24 |
| Leisure | -1.57 | 0.87 | -0.11 |
| Money | -0.34 | -1.26 | 1.38 |
| Health | -2.51 | -2.43 | 3.53 |
| Housing | 2.96 | 0.90 | -2.25 |
| Collective events | -1.72 | -0.67 | 1.45 |
| Other domain | -0.55 | 0.60 | -0.33 |
| Not clear: Poor information | 1.33 | -1.33 | 0.68 |
| Not clear: Multiple domains | 1.40 | -1.26 | 0.57 |

Negative values indicate that a certain event domain was mentioned less frequently in a certain age group (compared to the relative sample size of this group), whereas positive values indicate that an event domain was mentioned more frequently than expected in a certain age group.

### Event type

#### Table and statistical test

```
t3 <- table(data$event_type, data$age.theory)

kable(t3
      , caption="Frequencies of event type by age group"
      , escape=FALSE
      , label = NA) %>% 
  kable_styling(bootstrap_options = c("striped", "hover", "condensed"), 
                fixed_thead = T, full_width = FALSE, position="left")
```

Frequencies of event type by age group

|  | Young adulthood | Middle adulthood | Old adulthood |
| --- | --- | --- | --- |
| New job | 6 | 32 | 2 |
| End job | 1 | 10 | 11 |
| Work-related changes | 0 | 0 | 1 |
| Career milesone | 1 | 10 | 2 |
| Internship & voluntary | 1 | 0 | 2 |
| Retirement | 0 | 1 | 6 |
| Start education | 11 | 12 | 10 |
| Academic achievement | 16 | 19 | 6 |
| Academic failure | 3 | 1 | 0 |
| Change education | 0 | 2 | 0 |
| Engagement/marriage | 4 | 16 | 14 |
| Birth of own child | 4 | 14 | 0 |
| Pregnancy | 1 | 4 | 1 |
| New friendship | 1 | 2 | 2 |
| New romantic relationship | 3 | 6 | 5 |
| Get together | 0 | 6 | 28 |
| Improvement relationships | 0 | 4 | 3 |
| Problems in relationships | 0 | 2 | 1 |
| Child leaves home | 1 | 2 | 0 |
| Breakup/divorece | 1 | 14 | 3 |
| Friendship dissolution | 0 | 1 | 2 |
| Death of a loved one | 2 | 34 | 35 |
| Pet-related event | 3 | 8 | 7 |
| Vacation | 7 | 61 | 58 |
| Celebrating a special occasion | 4 | 38 | 44 |
| Change in lifestyle | 1 | 6 | 2 |
| Personal achievment | 1 | 7 | 10 |
| Financial difficulties | 0 | 0 | 1 |
| Imporvement in finances | 0 | 0 | 1 |
| Major investment | 1 | 4 | 4 |
| Selling a property | 0 | 0 | 3 |
| Illness or injury | 3 | 32 | 39 |
| Recovery from illness/injury | 0 | 9 | 15 |
| Infertility | 1 | 0 | 0 |
| Medical intervention | 1 | 5 | 31 |
| Relocation | 12 | 24 | 13 |
| Change in living situation | 0 | 6 | 1 |
| Collective event: war | 0 | 9 | 13 |
| Collective event: climate crisis | 0 | 0 | 0 |
| Collective event: pandemic | 0 | 11 | 23 |
| Collective event: other | 2 | 3 | 2 |
| Religious/Spiritual event | 0 | 0 | 0 |
| Legal troubles | 0 | 1 | 3 |
| Care duties | 0 | 0 | 2 |
| Miscarriage | 1 | 0 | 1 |
| Close other experienced childbirth | 4 | 16 | 32 |
| Other event | 1 | 6 | 8 |
| Not clear: Poor information | 3 | 8 | 20 |
| Not clear: More than one event | 4 | 10 | 16 |

```
## Check assumption
ratio.low.freq <- sum(chisq.test(table(data$event_type, data$age.theory))$expected <= 5)/length(chisq.test(table(data$event_type, data$age.theory))$expected)
# Fishers exact test recommended

## Results
fisher.test(table(data$event_type, data$age.theory), simulate.p.value = TRUE)
```

```
## 
##  Fisher's Exact Test for Count Data with simulated p-value (based on
##  2000 replicates)
## 
## data:  table(data$event_type, data$age.theory)
## p-value = 0.0004998
## alternative hypothesis: two.sided
```

The mentioned event types differ significantly between age groups.

#### Graph

```
ggplot(aes(x = event_type), data = data) +
  geom_bar(aes(fill = data$age.theory)) +
  labs(y = "Frequencies", x = "Event types") +
  theme_pub() +
  theme(axis.text.x = element_text(angle = 90, vjust = 0.5, hjust=1)) +
  theme(legend.text=element_text(size=13))
```

Relative frequencies:

```
prop <- as.data.frame(prop.table(table(data$event_type, data$age.theory), margin = 2))

p2 <- ggplot(aes(x = Var1, y = Freq), data = prop) +
  geom_col(stat = identity, aes(fill = Var2)) +
  labs(y = "Relative frequencies", x = "Event types") +
  theme_pub() +
  theme(axis.text.x = element_text(angle = 90, vjust = 0.5, hjust=1)) +
  theme(legend.text=element_text(size=13))

p2
```

#### Residuals

```
t4 <- round(chisq.test(table(data$event_type, data$age.theory))$residuals,2)

kable(t4
      , caption="Residuals of event types by age group"
      , escape=FALSE
      , label = NA) %>% 
  kable_styling(bootstrap_options = c("striped", "hover", "condensed"), 
                fixed_thead = T, full_width = FALSE, position="left")
```

Residuals of event types by age group

|  | Young adulthood | Middle adulthood | Old adulthood |
| --- | --- | --- | --- |
| New job | 0.99 | 3.48 | -3.84 |
| End job | -0.82 | 0.13 | 0.26 |
| Work-related changes | -0.32 | -0.66 | 0.79 |
| Career milesone | -0.27 | 1.81 | -1.64 |
| Internship & voluntary | 1.27 | -1.14 | 0.52 |
| Retirement | -0.84 | -1.18 | 1.53 |
| Start education | 4.22 | -0.64 | -1.35 |
| Academic achievement | 5.85 | 0.26 | -2.98 |
| Academic failure | 4.10 | -0.57 | -1.36 |
| Change education | -0.45 | 1.21 | -0.96 |
| Engagement/marriage | 0.31 | 0.30 | -0.44 |
| Birth of own child | 1.63 | 2.19 | -2.89 |
| Pregnancy | 0.51 | 0.85 | -1.07 |
| New friendship | 0.70 | -0.12 | -0.21 |
| New romantic relationship | 1.34 | -0.05 | -0.58 |
| Get together | -1.85 | -2.30 | 3.09 |
| Improvement relationships | -0.84 | 0.54 | -0.13 |
| Problems in relationships | -0.55 | 0.60 | -0.33 |
| Child leaves home | 1.27 | 0.60 | -1.18 |
| Breakup/divorece | -0.60 | 2.19 | -1.85 |
| Friendship dissolution | -0.55 | -0.27 | 0.52 |
| Death of a loved one | -1.92 | 0.54 | 0.38 |
| Pet-related event | 0.88 | 0.05 | -0.46 |
| Vacation | -1.59 | 0.80 | -0.04 |
| Celebrating a special occasion | -1.58 | 0.07 | 0.67 |
| Change in lifestyle | 0.10 | 1.04 | -1.06 |
| Personal achievment | -0.60 | -0.31 | 0.58 |
| Financial difficulties | -0.32 | -0.66 | 0.79 |
| Imporvement in finances | -0.32 | -0.66 | 0.79 |
| Major investment | 0.10 | 0.03 | -0.08 |
| Selling a property | -0.55 | -1.14 | 1.37 |
| Illness or injury | -1.63 | -0.06 | 0.81 |
| Recovery from illness/injury | -1.55 | -0.46 | 1.17 |
| Infertility | 2.84 | -0.66 | -0.68 |
| Medical intervention | -1.41 | -2.78 | 3.36 |
| Relocation | 3.19 | 0.56 | -2.03 |
| Change in living situation | -0.84 | 1.68 | -1.24 |
| Collective event: war | -1.49 | -0.20 | 0.88 |
| Collective event: climate crisis |  |  |  |
| Collective event: pandemic | -1.85 | -1.00 | 1.83 |
| Collective event: other | 1.54 | -0.03 | -0.69 |
| Religious/Spiritual event |  |  |  |
| Legal troubles | -0.63 | -0.57 | 0.84 |
| Care duties | -0.45 | -0.93 | 1.12 |
| Miscarriage | 1.78 | -0.93 | 0.08 |
| Close other experienced childbirth | -0.54 | -1.41 | 1.62 |
| Other event | -0.41 | -0.22 | 0.40 |
| Not clear: Poor information | -0.07 | -1.51 | 1.49 |
| Not clear: More than one event | 0.57 | -0.86 | 0.57 |

Negative values indicate that a certain event type was mentioned less frequently in a certain age group (compared to the relative sample size of this group), whereas positive values indicate that an event type was mentioned more frequently than expected in a certain age group.

## Quantile-based classification of age

### Event domains

#### Table and statistical test

```
t1 <- table(data$event_domain, data$age.terciles)

kable(t1
      , caption="Frequencies of event domains by age group"
      , escape=FALSE
      , label = NA) %>% 
  kable_styling(bootstrap_options = c("striped", "hover", "condensed"), 
                fixed_thead = T, full_width = FALSE, position="left")
```

Frequencies of event domains by age group

|  | Young | Middle | Old |
| --- | --- | --- | --- |
| Work | 44 | 41 | 8 |
| Education | 47 | 19 | 9 |
| Family | 72 | 99 | 82 |
| Romantic | 34 | 19 | 20 |
| Other social | 13 | 13 | 20 |
| Leisure | 62 | 76 | 61 |
| Money | 3 | 4 | 7 |
| Health | 21 | 47 | 77 |
| Housing | 32 | 12 | 8 |
| Collective events | 12 | 22 | 29 |
| Other domain | 0 | 2 | 1 |
| Not clear: Poor information | 3 | 2 | 3 |
| Not clear: Multiple domains | 5 | 10 | 5 |

```
## Check assumption
ratio.low.freq <- sum(chisq.test(table(data$event_domain, data$age.terciles))$expected <= 5)/length(chisq.test(table(data$event_domain, data$age.terciles))$expected)
# Fishers exact test recommended

## Results
fisher.test(table(data$event_domain, data$age.terciles), simulate.p.value = TRUE)
```

```
## 
##  Fisher's Exact Test for Count Data with simulated p-value (based on
##  2000 replicates)
## 
## data:  table(data$event_domain, data$age.terciles)
## p-value = 0.0004998
## alternative hypothesis: two.sided
```

The mentioned event domains differ significantly between age groups.

#### Graph

```
ggplot(aes(x = event_domain), data = data) +
  geom_bar(aes(fill = data$age.terciles)) +
  labs(y = "Frequencies", x = "Event domains") +
  theme_pub() +
  theme(axis.text.x = element_text(angle = 90, vjust = 0.5, hjust=1)) +
  theme(legend.text=element_text(size=13))
```

#### Residuals

```
t2 <- round(chisq.test(table(data$event_domain, data$age.terciles))$residuals,2)

kable(t2
      , caption="Residuals of event domains by age group"
      , escape=FALSE
      , label = NA) %>% 
  kable_styling(bootstrap_options = c("striped", "hover", "condensed"), 
                fixed_thead = T, full_width = FALSE, position="left")
```

Residuals of event domains by age group

|  | Young | Middle | Old |
| --- | --- | --- | --- |
| Work | 2.33 | 1.47 | -3.95 |
| Education | 4.40 | -1.42 | -3.02 |
| Family | -1.34 | 1.09 | 0.23 |
| Romantic | 1.96 | -1.30 | -0.64 |
| Other social | -0.60 | -0.78 | 1.43 |
| Leisure | -0.53 | 0.75 | -0.24 |
| Money | -0.77 | -0.41 | 1.22 |
| Health | -3.93 | -0.54 | 4.60 |
| Housing | 3.52 | -1.46 | -2.08 |
| Collective events | -1.96 | -0.02 | 2.04 |
| Other domain | -1.00 | 0.92 | 0.05 |
| Not clear: Poor information | 0.20 | -0.48 | 0.30 |
| Not clear: Multiple domains | -0.65 | 1.13 | -0.53 |

Negative values indicate that a certain event domain was mentioned less frequently in a certain age group (compared to the relative sample size of this group), whereas positive values indicate that an event domain was mentioned more frequently than expected in a certain age group.

### Event type

#### Table and statistical test

```
t3 <- table(data$event_type, data$age.terciles)

kable(t3
      , caption="Frequencies of event type by age group"
      , escape=FALSE
      , label = NA) %>% 
  kable_styling(bootstrap_options = c("striped", "hover", "condensed"), 
                fixed_thead = T, full_width = FALSE, position="left")
```

Frequencies of event type by age group

|  | Young | Middle | Old |
| --- | --- | --- | --- |
| New job | 25 | 14 | 1 |
| End job | 8 | 13 | 1 |
| Work-related changes | 0 | 1 | 0 |
| Career milesone | 7 | 5 | 1 |
| Internship & voluntary | 1 | 1 | 1 |
| Retirement | 0 | 6 | 1 |
| Start education | 20 | 8 | 5 |
| Academic achievement | 24 | 12 | 5 |
| Academic failure | 3 | 1 | 0 |
| Change education | 1 | 1 | 0 |
| Engagement/marriage | 12 | 16 | 6 |
| Birth of own child | 17 | 1 | 0 |
| Pregnancy | 5 | 0 | 1 |
| New friendship | 3 | 1 | 1 |
| New romantic relationship | 6 | 6 | 2 |
| Get together | 2 | 8 | 24 |
| Improvement relationships | 1 | 4 | 2 |
| Problems in relationships | 2 | 0 | 1 |
| Child leaves home | 2 | 1 | 0 |
| Breakup/divorece | 13 | 3 | 2 |
| Friendship dissolution | 0 | 2 | 1 |
| Death of a loved one | 14 | 37 | 20 |
| Pet-related event | 6 | 11 | 1 |
| Vacation | 38 | 48 | 40 |
| Celebrating a special occasion | 28 | 20 | 38 |
| Change in lifestyle | 5 | 2 | 2 |
| Personal achievment | 5 | 8 | 5 |
| Financial difficulties | 0 | 0 | 1 |
| Imporvement in finances | 0 | 0 | 1 |
| Major investment | 4 | 3 | 2 |
| Selling a property | 0 | 0 | 3 |
| Illness or injury | 13 | 30 | 31 |
| Recovery from illness/injury | 5 | 4 | 15 |
| Infertility | 1 | 0 | 0 |
| Medical intervention | 3 | 10 | 24 |
| Relocation | 29 | 12 | 8 |
| Change in living situation | 4 | 2 | 1 |
| Collective event: war | 4 | 9 | 9 |
| Collective event: climate crisis | 0 | 0 | 0 |
| Collective event: pandemic | 5 | 11 | 18 |
| Collective event: other | 4 | 1 | 2 |
| Religious/Spiritual event | 0 | 0 | 0 |
| Legal troubles | 0 | 3 | 1 |
| Care duties | 0 | 1 | 1 |
| Miscarriage | 1 | 0 | 1 |
| Close other experienced childbirth | 9 | 24 | 19 |
| Other event | 6 | 2 | 7 |
| Not clear: Poor information | 5 | 10 | 16 |
| Not clear: More than one event | 7 | 14 | 9 |

```
## Check assumption
ratio.low.freq <- sum(chisq.test(table(data$event_type, data$age.terciles))$expected <= 5)/length(chisq.test(table(data$event_type, data$age.terciles))$expected)
# Fishers exact test recommended

## Results
fisher.test(table(data$event_type, data$age.terciles), simulate.p.value = TRUE)
```

```
## 
##  Fisher's Exact Test for Count Data with simulated p-value (based on
##  2000 replicates)
## 
## data:  table(data$event_type, data$age.terciles)
## p-value = 0.0004998
## alternative hypothesis: two.sided
```

The mentioned event types differ significantly between age groups.

#### Graph

```
ggplot(aes(x = event_type), data = data) +
  geom_bar(aes(fill = data$age.terciles)) +
  labs(y = "Frequencies", x = "Event types") +
  theme_pub() +
  theme(axis.text.x = element_text(angle = 90, vjust = 0.5, hjust=1)) +
  theme(legend.text=element_text(size=13))
```

#### Residuals

```
t4 <- round(chisq.test(table(data$event_type, data$age.terciles))$residuals,2)

kable(t4
      , caption="Residuals of event types by age group"
      , escape=FALSE
      , label = NA) %>% 
  kable_styling(bootstrap_options = c("striped", "hover", "condensed"), 
                fixed_thead = T, full_width = FALSE, position="left")
```

Residuals of event types by age group

|  | Young | Middle | Old |
| --- | --- | --- | --- |
| New job | 3.20 | -0.01 | -3.27 |
| End job | 0.25 | 1.90 | -2.26 |
| Work-related changes | -0.58 | 1.10 | -0.56 |
| Career milesone | 1.28 | 0.21 | -1.53 |
| Internship & voluntary | 0.00 | -0.05 | 0.05 |
| Retirement | -1.53 | 2.26 | -0.82 |
| Start education | 2.71 | -1.05 | -1.68 |
| Academic achievement | 2.80 | -0.63 | -2.21 |
| Academic failure | 1.44 | -0.34 | -1.12 |
| Change education | 0.41 | 0.36 | -0.80 |
| Engagement/marriage | 0.20 | 1.18 | -1.45 |
| Birth of own child | 4.49 | -2.11 | -2.39 |
| Pregnancy | 2.12 | -1.45 | -0.65 |
| New friendship | 1.03 | -0.57 | -0.46 |
| New romantic relationship | 0.62 | 0.49 | -1.15 |
| Get together | -2.77 | -1.14 | 4.04 |
| Improvement relationships | -0.87 | 0.99 | -0.14 |
| Problems in relationships | 1.00 | -1.03 | 0.05 |
| Child leaves home | 1.00 | -0.05 | -0.97 |
| Breakup/divorece | 2.86 | -1.32 | -1.55 |
| Friendship dissolution | -1.00 | 0.92 | 0.05 |
| Death of a loved one | -1.99 | 2.43 | -0.52 |
| Pet-related event | 0.00 | 1.87 | -1.97 |
| Vacation | -0.62 | 0.58 | 0.03 |
| Celebrating a special occasion | -0.12 | -1.85 | 2.07 |
| Change in lifestyle | 1.15 | -0.65 | -0.50 |
| Personal achievment | -0.41 | 0.67 | -0.29 |
| Financial difficulties | -0.58 | -0.59 | 1.22 |
| Imporvement in finances | -0.58 | -0.59 | 1.22 |
| Major investment | 0.58 | -0.09 | -0.50 |
| Selling a property | -1.00 | -1.03 | 2.11 |
| Illness or injury | -2.35 | 0.80 | 1.57 |
| Recovery from illness/injury | -1.06 | -1.52 | 2.69 |
| Infertility | 1.15 | -0.59 | -0.56 |
| Medical intervention | -2.66 | -0.82 | 3.60 |
| Relocation | 3.13 | -1.25 | -1.90 |
| Change in living situation | 1.09 | -0.29 | -0.82 |
| Collective event: war | -1.23 | 0.46 | 0.78 |
| Collective event: climate crisis |  |  |  |
| Collective event: pandemic | -1.88 | -0.27 | 2.21 |
| Collective event: other | 1.09 | -0.93 | -0.14 |
| Religious/Spiritual event |  |  |  |
| Legal troubles | -1.15 | 1.35 | -0.24 |
| Care duties | -0.82 | 0.36 | 0.46 |
| Miscarriage | 0.41 | -0.84 | 0.46 |
| Close other experienced childbirth | -2.00 | 1.35 | 0.63 |
| Other event | 0.45 | -1.42 | 1.04 |
| Not clear: Poor information | -1.66 | -0.26 | 1.98 |
| Not clear: More than one event | -0.95 | 1.07 | -0.16 |

Negative values indicate that a certain event type was mentioned less frequently in a certain age group (compared to the relative sample size of this group), whereas positive values indicate that an event type was mentioned more frequently than expected in a certain age group.

# Section 3: Research Question 2 - Perception of the most important life event across age

## Age (metric)

### No covariate

#### Table

```
gam_chall <- gam(ecq.chall ~ s(age), data = data, method = "REML")
res_chall <- c(round(summary(gam_chall)$edf,2), round(summary(gam_chall)$s.pv,3), 
               round(summary(gam_chall)$r.sq,3))
gam_changeww <- gam(ecq.changeww ~ s(age), data = data, method = "REML")
res_changeww <- c(round(summary(gam_changeww)$edf,2), round(summary(gam_changeww)$s.pv,3), 
               round(summary(gam_changeww)$r.sq,3))

gam_emosig <- gam(ecq.emosig ~ s(age), data = data, method = "REML")
res_emosig <- c(round(summary(gam_emosig)$edf,2), round(summary(gam_emosig)$s.pv,3), 
               round(summary(gam_emosig)$r.sq,3))

gam_extrao <- gam(ecq.extrao ~ s(age), data = data, method = "REML")
res_extrao <- c(round(summary(gam_extrao)$edf,2), round(summary(gam_extrao)$s.pv,3), 
               round(summary(gam_extrao)$r.sq,3))

gam_extcon <- gam(ecq.extcon ~ s(age), data = data, method = "REML")
res_extcon <- c(round(summary(gam_extcon)$edf,2), round(summary(gam_extcon)$s.pv,3), 
               round(summary(gam_extcon)$r.sq,3))

gam_impact <- gam(ecq.impact ~ s(age), data = data, method = "REML")
res_impact <- c(round(summary(gam_impact)$edf,2), round(summary(gam_impact)$s.pv,3), 
               round(summary(gam_impact)$r.sq,3))

gam_predict <- gam(ecq.predict ~ s(age), data = data, method = "REML")
res_predict <- c(round(summary(gam_predict)$edf,2), round(summary(gam_predict)$s.pv,3), 
               round(summary(gam_predict)$r.sq,3))

gam_socstat <- gam(ecq.socstat ~ s(age), data = data, method = "REML")
res_socstat <- c(round(summary(gam_socstat)$edf,2), round(summary(gam_socstat)$s.pv,3), 
               round(summary(gam_socstat)$r.sq,3))

gam_val <- gam(ecq.val ~ s(age), data = data, method = "REML")
res_val <- c(round(summary(gam_val)$edf,2), round(summary(gam_val)$s.pv,3), 
               round(summary(gam_val)$r.sq,3))

## Combine results
res_gam <- as.data.frame(rbind(res_chall, res_changeww, res_emosig,
                               res_extcon, res_extrao, res_impact,
                               res_predict, res_socstat, res_val))
names(res_gam) <- c("Edf", "p", "R^2^")
row.names(res_gam) <- c("Challenge", "Change in world views", 
                        "Emotional significance", "External control", 
                        "Extraordinariness", "Impact",
                        "Predictability", "Social status change", 
                        "Valence")

kable(res_gam
      , caption="Results of GAMs with a smoothed age-term as predictor"
      , escape=FALSE
      , label = NA) %>% 
  kable_styling(bootstrap_options = c("striped", "hover"), 
                fixed_thead = T, full_width = FALSE, position="left")
```

Results of GAMs with a smoothed age-term as predictor

|  | Edf | p | R2 |
| --- | --- | --- | --- |
| Challenge | 3.42 | 0.019 | 0.011 |
| Change in world views | 1.00 | 0.000 | 0.020 |
| Emotional significance | 2.03 | 0.347 | 0.002 |
| External control | 1.35 | 0.007 | 0.009 |
| Extraordinariness | 1.00 | 0.187 | 0.001 |
| Impact | 2.46 | 0.000 | 0.021 |
| Predictability | 4.00 | 0.009 | 0.014 |
| Social status change | 7.63 | 0.000 | 0.044 |
| Valence | 2.65 | 0.001 | 0.016 |

#### Graph: Challenge

```
g1 <- plot(gam_chall, shift = coef(gam_chall)[1], shade = TRUE, shade.col = "lightblue", xlab = "Age", ylab = "Challenge", resiudals = TRUE, cex.lab = 1.5, cex.axis = 1.5)
```

#### Graph: Change in world views

```
g2 <- plot(gam_changeww, shift = coef(gam_changeww)[1], shade = TRUE, shade.col = "lightblue", xlab = "Age", ylab = "Change in world views", cex.lab = 1.5, cex.axis = 1.5)
```

#### Graph: Emotional signifiance

```
g3 <- plot(gam_emosig, shift = coef(gam_emosig)[1], shade = TRUE, shade.col = "lightblue", xlab = "Age", ylab = "Emotional significance", cex.lab = 1.5, cex.axis = 1.5)
```

#### Graph: External control

```
g4 <- plot(gam_extcon, shift = coef(gam_extcon)[1], shade = TRUE, shade.col = "lightblue", xlab = "Age", ylab = "External control", cex.lab = 1.5, cex.axis = 1.5)
```

#### Graph: Extraordinariness

```
g5 <- plot(gam_extrao, shift = coef(gam_extrao)[1], shade = TRUE, shade.col = "lightblue", xlab = "Age", ylab = "Extraordinariness", cex.lab = 1.5, cex.axis = 1.5)
```

#### Graph: Impact

```
g6 <- plot(gam_impact, shift = coef(gam_impact)[1], shade = TRUE, shade.col = "lightblue", xlab = "Age", ylab = "Impact", cex.lab = 1.5, cex.axis = 1.5)
```

#### Graph: Predictability

```
g7 <- plot(gam_predict, shift = coef(gam_predict)[1], shade = TRUE, shade.col = "lightblue", xlab = "Age", ylab = "Predictability", cex.lab = 1.5, cex.axis = 1.5)
```

#### Graph: Social status change

```
g8 <- plot(gam_socstat, shift = coef(gam_socstat)[1], shade = TRUE, shade.col = "lightblue", xlab = "Age", ylab = "Social status change", cex.lab = 1.5, cex.axis = 1.5)
```

#### Graph: Valence

```
g9 <- plot(gam_val, shift = coef(gam_val)[1], shade = TRUE, shade.col = "lightblue", xlab = "Age", ylab = "Valence", cex.lab = 1.5, cex.axis = 1.5)
```

### Event type as covariate

#### Tables

```
contrasts(data$event_type) <- contr.wec(data$event_type, omitted = "Vacation") #Code event type using 

gam_chall <- gam(ecq.chall ~ s(age) + event_type, data = data, method = "REML")
res_chall <- c(round(summary(gam_chall)$edf,2), round(summary(gam_chall)$s.pv,3), 
               round(summary(gam_chall)$r.sq,3))

gam_changeww <- gam(ecq.changeww ~ s(age) + event_type, data = data, method = "REML")
res_changeww <- c(round(summary(gam_changeww)$edf,2), round(summary(gam_changeww)$s.pv,3), 
               round(summary(gam_changeww)$r.sq,3))

gam_emosig <- gam(ecq.emosig ~ s(age) + event_type, data = data, method = "REML")
res_emosig <- c(round(summary(gam_emosig)$edf,2), round(summary(gam_emosig)$s.pv,3), 
               round(summary(gam_emosig)$r.sq,3))

gam_extrao <- gam(ecq.extrao ~ s(age) + event_type, data = data, method = "REML")
res_extrao <- c(round(summary(gam_extrao)$edf,2), round(summary(gam_extrao)$s.pv,3), 
               round(summary(gam_extrao)$r.sq,3))

gam_extcon <- gam(ecq.extcon ~ s(age) + event_type, data = data, method = "REML")
res_extcon <- c(round(summary(gam_extcon)$edf,2), round(summary(gam_extcon)$s.pv,3), 
               round(summary(gam_extcon)$r.sq,3))

gam_impact <- gam(ecq.impact ~ s(age) + event_type, data = data, method = "REML")
res_impact <- c(round(summary(gam_impact)$edf,2), round(summary(gam_impact)$s.pv,3), 
               round(summary(gam_impact)$r.sq,3))

gam_predict <- gam(ecq.predict ~ s(age) + event_type, data = data, method = "REML")
res_predict <- c(round(summary(gam_predict)$edf,2), round(summary(gam_predict)$s.pv,3), 
               round(summary(gam_predict)$r.sq,3))

gam_socstat <- gam(ecq.socstat ~ s(age) + event_type, data = data, method = "REML")
res_socstat <- c(round(summary(gam_socstat)$edf,2), round(summary(gam_socstat)$s.pv,3), 
               round(summary(gam_socstat)$r.sq,3))

gam_val <- gam(ecq.val ~ s(age) + event_type, data = data, method = "REML")
res_val <- c(round(summary(gam_val)$edf,2), round(summary(gam_val)$s.pv,3), 
               round(summary(gam_val)$r.sq,3))

## Combine results
res_gam <- as.data.frame(rbind(res_chall, res_changeww, res_emosig,
                               res_extcon, res_extrao, res_impact,
                               res_predict, res_socstat, res_val))
names(res_gam) <- c("Edf", "p", "R^2^")
row.names(res_gam) <- c("Challenge", "Change in world views", 
                        "Emotional significance", "External control", 
                        "Extraordinariness", "Impact",
                        "Predictability", "Social status change", 
                        "Valence")

kable(res_gam
      , caption="Results of GAMs with a smoothed age-term as predictor and event type as covariate"
      , escape=FALSE
      , label = NA) %>% 
  kable_styling(bootstrap_options = c("striped", "hover"), 
                fixed_thead = T, full_width = FALSE, position="left")
```

Results of GAMs with a smoothed age-term as predictor and event type as covariate

|  | Edf | p | R2 |
| --- | --- | --- | --- |
| Challenge | 2.43 | 0.007 | 0.326 |
| Change in world views | 1.00 | 0.000 | 0.170 |
| Emotional significance | 2.12 | 0.463 | 0.085 |
| External control | 1.00 | 0.008 | 0.162 |
| Extraordinariness | 1.00 | 0.201 | 0.100 |
| Impact | 2.36 | 0.001 | 0.145 |
| Predictability | 1.01 | 0.713 | 0.362 |
| Social status change | 6.42 | 0.011 | 0.151 |
| Valence | 2.06 | 0.276 | 0.565 |

#### Graph: Challenge

```
plot(gam_chall, shift = coef(gam_chall)[1], shade = TRUE, shade.col = "lightblue", xlab = "Age", ylab = "Challenge", resiudals = TRUE, cex.lab = 1.5, cex.axis = 1.5)
```

#### Graph: Change in world views

```
plot(gam_changeww, shift = coef(gam_changeww)[1], shade = TRUE, shade.col = "lightblue", xlab = "Age", ylab = "Change in world views", cex.lab = 1.5, cex.axis = 1.5)
```

#### Graph: Emotional signifiance

```
plot(gam_emosig, shift = coef(gam_emosig)[1], shade = TRUE, shade.col = "lightblue", xlab = "Age", ylab = "Emotional significance", cex.lab = 1.5, cex.axis = 1.5)
```

#### Graph: External control

```
plot(gam_extcon, shift = coef(gam_extcon)[1], shade = TRUE, shade.col = "lightblue", xlab = "Age", ylab = "External control", cex.lab = 1.5, cex.axis = 1.5)
```

#### Graph: Extraordinariness

```
plot(gam_extrao, shift = coef(gam_extrao)[1], shade = TRUE, shade.col = "lightblue", xlab = "Age", ylab = "Extraordinariness", cex.lab = 1.5, cex.axis = 1.5)
```

#### Graph: Impact

```
plot(gam_impact, shift = coef(gam_impact)[1], shade = TRUE, shade.col = "lightblue", xlab = "Age", ylab = "Impact", cex.lab = 1.5, cex.axis = 1.5)
```

#### Graph: Predictability

```
plot(gam_predict, shift = coef(gam_predict)[1], shade = TRUE, shade.col = "lightblue", xlab = "Age", ylab = "Predictability", cex.lab = 1.5, cex.axis = 1.5)
```

#### Graph: Social status change

```
plot(gam_socstat, shift = coef(gam_socstat)[1], shade = TRUE, shade.col = "lightblue", xlab = "Age", ylab = "Social status change", cex.lab = 1.5, cex.axis = 1.5)
```

#### Graph: Valence

```
plot(gam_val, shift = coef(gam_val)[1], shade = TRUE, shade.col = "lightblue", xlab = "Age", ylab = "Valence", cex.lab = 1.5, cex.axis = 1.5)
```

## Age (groups)

### Literature-based classification of age

#### Omnibus test

```
# Function to extract overall p-value of model (source: https://www.statology.org/r-extract-p-value-from-lm/)
overall_p <- function(my_model) {
    f <- summary(my_model)$fstatistic
    p <- pf(f[1],f[2],f[3],lower.tail=F)
    attributes(p) <- NULL
    return(p)
}

aov_chall <- summary(lm(data$ecq.chall ~ data$age.theory))
aov_chall_res <- c(round(aov_chall$fstatistic["value"],2), 
                   aov_chall$fstatistic["numdf"], 
                   aov_chall$fstatistic["dendf"], 
                   round(overall_p(lm(data$ecq.chall ~ data$age.theory)),3),
                   round(aov_chall$r.squared,3))

aov_changeww <- summary(lm(data$ecq.changeww ~ data$age.theory))
aov_changeww_res <- c(round(aov_changeww$fstatistic["value"],2), 
                   aov_changeww$fstatistic["numdf"], 
                   aov_changeww$fstatistic["dendf"], 
                   round(overall_p(lm(data$ecq.changeww ~ data$age.theory)),3),
                   round(aov_changeww$r.squared,3))

aov_emosig <- summary(lm(data$ecq.emosig ~ data$age.theory))
aov_emosig_res <- c(round(aov_emosig$fstatistic["value"],2), 
                   aov_emosig$fstatistic["numdf"], 
                   aov_emosig$fstatistic["dendf"], 
                   round(overall_p(lm(data$ecq.emosig ~ data$age.theory)),3),
                   round(aov_emosig$r.squared,3))

aov_extcon <- summary(lm(data$ecq.extcon ~ data$age.theory))
aov_extcon_res <- c(round(aov_extcon$fstatistic["value"],2), 
                   aov_extcon$fstatistic["numdf"], 
                   aov_extcon$fstatistic["dendf"], 
                   round(overall_p(lm(data$ecq.extcon ~ data$age.theory)),3),
                   round(aov_extcon$r.squared,3))

aov_extrao <- summary(lm(data$ecq.extrao ~ data$age.theory))
aov_extrao_res <- c(round(aov_extrao$fstatistic["value"],2), 
                   aov_extrao$fstatistic["numdf"], 
                   aov_extrao$fstatistic["dendf"], 
                   round(overall_p(lm(data$ecq.extrao ~ data$age.theory)),3),
                   round(aov_extrao$r.squared,3))

aov_impact <- summary(lm(data$ecq.impact ~ data$age.theory))
aov_impact_res <- c(round(aov_impact$fstatistic["value"],2), 
                   aov_impact$fstatistic["numdf"], 
                   aov_impact$fstatistic["dendf"], 
                   round(overall_p(lm(data$ecq.impact ~ data$age.theory)),3),
                   round(aov_impact$r.squared,3))

aov_predict <- summary(lm(data$ecq.predict ~ data$age.theory))
aov_predict_res <- c(round(aov_predict$fstatistic["value"],2), 
                   aov_predict$fstatistic["numdf"], 
                   aov_predict$fstatistic["dendf"], 
                   round(overall_p(lm(data$ecq.predict ~ data$age.theory)),3),
                   round(aov_predict$r.squared,3))

aov_socstat <- summary(lm(data$ecq.socstat ~ data$age.theory))
aov_socstat_res <- c(round(aov_socstat$fstatistic["value"],2), 
                   aov_socstat$fstatistic["numdf"], 
                   aov_socstat$fstatistic["dendf"], 
                   round(overall_p(lm(data$ecq.socstat ~ data$age.theory)),3),
                   round(aov_socstat$r.squared,3))

aov_val <- summary(lm(data$ecq.val ~ data$age.theory))
aov_val_res <- c(round(aov_val$fstatistic["value"],2), 
                   aov_val$fstatistic["numdf"], 
                   aov_val$fstatistic["dendf"], 
                   round(overall_p(lm(data$ecq.val ~ data$age.theory)),3),
                   round(aov_val$r.squared,3))

aov_res <- as.data.frame(rbind(aov_chall_res, aov_changeww_res, aov_emosig_res,
                 aov_extcon_res, aov_extrao_res, aov_impact_res,
                 aov_predict_res, aov_socstat_res, aov_val_res))

names(aov_res) <- c("F", "df1", "df2", "p", "R^2^")
row.names(aov_res) <- c("Challenge", "Change in world views", 
                        "Emotional significance", "External control", 
                        "Extraordinariness", "Impact",
                        "Predictability", "Social status change", 
                        "Valence")

kable(aov_res
      , caption="Results of anova with age group as predictor"
      , escape=FALSE
      , label = NA) %>% 
  kable_styling(bootstrap_options = c("striped", "hover"), 
                fixed_thead = T, full_width = FALSE, position="left")
```

Results of anova with age group as predictor

|  | F | df1 | df2 | p | R2 |
| --- | --- | --- | --- | --- | --- |
| Challenge | 4.39 | 2 | 1036 | 0.013 | 0.008 |
| Change in world views | 11.40 | 2 | 1036 | 0.000 | 0.022 |
| Emotional significance | 0.59 | 2 | 1039 | 0.554 | 0.001 |
| External control | 3.98 | 2 | 1030 | 0.019 | 0.008 |
| Extraordinariness | 0.60 | 2 | 993 | 0.548 | 0.001 |
| Impact | 9.49 | 2 | 1037 | 0.000 | 0.018 |
| Predictability | 2.70 | 2 | 1033 | 0.068 | 0.005 |
| Social status change | 1.13 | 2 | 1034 | 0.324 | 0.002 |
| Valence | 6.65 | 2 | 1035 | 0.001 | 0.013 |

#### Post-hoc comparisons

Challenge:

```
model_chall <- lm(ecq.chall ~ age.theory, data = data)
emm_chall <- emmeans(model_chall, specs = "age.theory")
pairs(emm_chall)
```

```
##  contrast                           estimate    SE   df t.ratio p.value
##  Young adulthood - Middle adulthood  -0.0521 0.171 1036  -0.304  0.9504
##  Young adulthood - Old adulthood      0.2481 0.171 1036   1.454  0.3138
##  Middle adulthood - Old adulthood     0.3001 0.104 1036   2.896  0.0108
## 
## P value adjustment: tukey method for comparing a family of 3 estimates
```

Change in world views:

```
model_changeww <- lm(ecq.changeww ~ age.theory, data = data)
emm_changeww <- emmeans(model_changeww, specs = "age.theory")
pairs(emm_changeww)
```

```
##  contrast                           estimate     SE   df t.ratio p.value
##  Young adulthood - Middle adulthood    0.323 0.1550 1036   2.084  0.0936
##  Young adulthood - Old adulthood       0.644 0.1543 1036   4.176  0.0001
##  Middle adulthood - Old adulthood      0.321 0.0933 1036   3.441  0.0017
## 
## P value adjustment: tukey method for comparing a family of 3 estimates
```

External control:

```
model_extcon <- lm(ecq.extcon ~ age.theory, data = data)
emm_extcon <- emmeans(model_extcon, specs = "age.theory")
pairs(emm_extcon)
```

```
##  contrast                           estimate    SE   df t.ratio p.value
##  Young adulthood - Middle adulthood   0.0701 0.177 1030   0.396  0.9173
##  Young adulthood - Old adulthood     -0.2283 0.176 1030  -1.294  0.3987
##  Middle adulthood - Old adulthood    -0.2984 0.107 1030  -2.778  0.0153
## 
## P value adjustment: tukey method for comparing a family of 3 estimates
```

Impact:

```
model_impact <- lm(ecq.impact ~ age.theory, data = data)
emm_impact <- emmeans(model_impact, specs = "age.theory")
pairs(emm_impact)
```

```
##  contrast                           estimate     SE   df t.ratio p.value
##  Young adulthood - Middle adulthood    0.526 0.1463 1037   3.593  0.0010
##  Young adulthood - Old adulthood       0.634 0.1455 1037   4.356  <.0001
##  Middle adulthood - Old adulthood      0.108 0.0884 1037   1.225  0.4385
## 
## P value adjustment: tukey method for comparing a family of 3 estimates
```

Valence:

```
model_val <- lm(ecq.val ~ age.theory, data = data)
emm_val <- emmeans(model_val, specs = "age.theory")
pairs(emm_val)
```

```
##  contrast                           estimate    SE   df t.ratio p.value
##  Young adulthood - Middle adulthood   0.5815 0.175 1035   3.330  0.0026
##  Young adulthood - Old adulthood      0.6222 0.174 1035   3.577  0.0011
##  Middle adulthood - Old adulthood     0.0406 0.106 1035   0.385  0.9217
## 
## P value adjustment: tukey method for comparing a family of 3 estimates
```

### Quantile-based classification of age

#### Omnibus test

```
# Function to extract overall p-value of model (source: https://www.statology.org/r-extract-p-value-from-lm/)
overall_p <- function(my_model) {
    f <- summary(my_model)$fstatistic
    p <- pf(f[1],f[2],f[3],lower.tail=F)
    attributes(p) <- NULL
    return(p)
}

aov_chall <- summary(lm(data$ecq.chall ~ data$age.terciles))
aov_chall_res <- c(round(aov_chall$fstatistic["value"],2), 
                   aov_chall$fstatistic["numdf"], 
                   aov_chall$fstatistic["dendf"], 
                   round(overall_p(lm(data$ecq.chall ~ data$age.terciles)),3),
                   round(aov_chall$r.squared,3))

aov_changeww <- summary(lm(data$ecq.changeww ~ data$age.terciles))
aov_changeww_res <- c(round(aov_changeww$fstatistic["value"],2), 
                   aov_changeww$fstatistic["numdf"], 
                   aov_changeww$fstatistic["dendf"], 
                   round(overall_p(lm(data$ecq.changeww ~ data$age.terciles)),3),
                   round(aov_changeww$r.squared,3))

aov_emosig <- summary(lm(data$ecq.emosig ~ data$age.terciles))
aov_emosig_res <- c(round(aov_emosig$fstatistic["value"],2), 
                   aov_emosig$fstatistic["numdf"], 
                   aov_emosig$fstatistic["dendf"], 
                   round(overall_p(lm(data$ecq.emosig ~ data$age.terciles)),3),
                   round(aov_emosig$r.squared,3))

aov_extcon <- summary(lm(data$ecq.extcon ~ data$age.terciles))
aov_extcon_res <- c(round(aov_extcon$fstatistic["value"],2), 
                   aov_extcon$fstatistic["numdf"], 
                   aov_extcon$fstatistic["dendf"], 
                   round(overall_p(lm(data$ecq.extcon ~ data$age.terciles)),3),
                   round(aov_extcon$r.squared,3))

aov_extrao <- summary(lm(data$ecq.extrao ~ data$age.terciles))
aov_extrao_res <- c(round(aov_extrao$fstatistic["value"],2), 
                   aov_extrao$fstatistic["numdf"], 
                   aov_extrao$fstatistic["dendf"], 
                   round(overall_p(lm(data$ecq.extrao ~ data$age.terciles)),3),
                   round(aov_extrao$r.squared,3))

aov_impact <- summary(lm(data$ecq.impact ~ data$age.terciles))
aov_impact_res <- c(round(aov_impact$fstatistic["value"],2), 
                   aov_impact$fstatistic["numdf"], 
                   aov_impact$fstatistic["dendf"], 
                   round(overall_p(lm(data$ecq.impact ~ data$age.terciles)),3),
                   round(aov_impact$r.squared,3))

aov_predict <- summary(lm(data$ecq.predict ~ data$age.terciles))
aov_predict_res <- c(round(aov_predict$fstatistic["value"],2), 
                   aov_predict$fstatistic["numdf"], 
                   aov_predict$fstatistic["dendf"], 
                   round(overall_p(lm(data$ecq.predict ~ data$age.terciles)),3),
                   round(aov_predict$r.squared,3))

aov_socstat <- summary(lm(data$ecq.socstat ~ data$age.terciles))
aov_socstat_res <- c(round(aov_socstat$fstatistic["value"],2), 
                   aov_socstat$fstatistic["numdf"], 
                   aov_socstat$fstatistic["dendf"], 
                   round(overall_p(lm(data$ecq.socstat ~ data$age.terciles)),3),
                   round(aov_socstat$r.squared,3))

aov_val <- summary(lm(data$ecq.val ~ data$age.terciles))
aov_val_res <- c(round(aov_val$fstatistic["value"],2), 
                   aov_val$fstatistic["numdf"], 
                   aov_val$fstatistic["dendf"], 
                   round(overall_p(lm(data$ecq.val ~ data$age.terciles)),3),
                   round(aov_val$r.squared,3))

aov_res <- as.data.frame(rbind(aov_chall_res, aov_changeww_res, aov_emosig_res,
                 aov_extcon_res, aov_extrao_res, aov_impact_res,
                 aov_predict_res, aov_socstat_res, aov_val_res))

names(aov_res) <- c("F", "df1", "df2", "p", "R^2^")
row.names(aov_res) <- c("Challenge", "Change in world views", 
                        "Emotional significance", "External control", 
                        "Extraordinariness", "Impact",
                        "Predictability", "Social status change", 
                        "Valence")

kable(aov_res
      , caption="Results of anova with age group as predictor"
      , escape=FALSE
      , label = NA) %>% 
  kable_styling(bootstrap_options = c("striped", "hover"), 
                fixed_thead = T, full_width = FALSE, position="left")
```

Results of anova with age group as predictor

|  | F | df1 | df2 | p | R2 |
| --- | --- | --- | --- | --- | --- |
| Challenge | 3.12 | 2 | 1036 | 0.044 | 0.006 |
| Change in world views | 10.69 | 2 | 1036 | 0.000 | 0.020 |
| Emotional significance | 1.94 | 2 | 1039 | 0.144 | 0.004 |
| External control | 5.48 | 2 | 1030 | 0.004 | 0.011 |
| Extraordinariness | 1.71 | 2 | 993 | 0.181 | 0.003 |
| Impact | 6.68 | 2 | 1037 | 0.001 | 0.013 |
| Predictability | 5.99 | 2 | 1033 | 0.003 | 0.011 |
| Social status change | 3.33 | 2 | 1034 | 0.036 | 0.006 |
| Valence | 6.39 | 2 | 1035 | 0.002 | 0.012 |

#### Post-hoc comparisons

Challenge:

```
model_chall <- lm(ecq.chall ~ age.terciles, data = data)
emm_chall <- emmeans(model_chall, specs = "age.terciles")
pairs(emm_chall)
```

```
##  contrast       estimate    SE   df t.ratio p.value
##  Young - Middle  0.25849 0.119 1036   2.175  0.0760
##  Young - Old     0.26263 0.122 1036   2.151  0.0803
##  Middle - Old    0.00413 0.121 1036   0.034  0.9994
## 
## P value adjustment: tukey method for comparing a family of 3 estimates
```

Change in world views:

```
model_changeww <- lm(ecq.changeww ~ age.terciles, data = data)
emm_changeww <- emmeans(model_changeww, specs = "age.terciles")
pairs(emm_changeww)
```

```
##  contrast       estimate    SE   df t.ratio p.value
##  Young - Middle    0.221 0.107 1036   2.059  0.0990
##  Young - Old       0.507 0.110 1036   4.616  <.0001
##  Middle - Old      0.286 0.109 1036   2.635  0.0232
## 
## P value adjustment: tukey method for comparing a family of 3 estimates
```

External control:

```
model_extcon <- lm(ecq.extcon ~ age.terciles, data = data)
emm_extcon <- emmeans(model_extcon, specs = "age.terciles")
pairs(emm_extcon)
```

```
##  contrast       estimate    SE   df t.ratio p.value
##  Young - Middle  -0.0303 0.123 1030  -0.247  0.9670
##  Young - Old     -0.3763 0.126 1030  -2.987  0.0081
##  Middle - Old    -0.3460 0.125 1030  -2.773  0.0156
## 
## P value adjustment: tukey method for comparing a family of 3 estimates
```

Impact:

```
model_impact <- lm(ecq.impact ~ age.terciles, data = data)
emm_impact <- emmeans(model_impact, specs = "age.terciles")
pairs(emm_impact)
```

```
##  contrast       estimate    SE   df t.ratio p.value
##  Young - Middle    0.203 0.102 1037   1.996  0.1136
##  Young - Old       0.380 0.104 1037   3.650  0.0008
##  Middle - Old      0.177 0.103 1037   1.723  0.1972
## 
## P value adjustment: tukey method for comparing a family of 3 estimates
```

Predictability:

```
model_predict <- lm(ecq.predict ~ age.terciles, data = data)
emm_predict <- emmeans(model_predict, specs = "age.terciles")
pairs(emm_predict)
```

```
##  contrast       estimate    SE   df t.ratio p.value
##  Young - Middle    0.304 0.119 1033   2.544  0.0298
##  Young - Old       0.405 0.123 1033   3.300  0.0029
##  Middle - Old      0.101 0.121 1033   0.833  0.6827
## 
## P value adjustment: tukey method for comparing a family of 3 estimates
```

Social status change:

```
model_socstat <- lm(ecq.socstat ~ age.terciles, data = data)
emm_socstat <- emmeans(model_socstat, specs = "age.terciles")
pairs(emm_socstat)
```

```
##  contrast       estimate     SE   df t.ratio p.value
##  Young - Middle  -0.1096 0.0531 1034  -2.065  0.0978
##  Young - Old     -0.1291 0.0545 1034  -2.371  0.0471
##  Middle - Old    -0.0195 0.0538 1034  -0.362  0.9304
## 
## P value adjustment: tukey method for comparing a family of 3 estimates
```

Valence:

```
model_val <- lm(ecq.val ~ age.terciles, data = data)
emm_val <- emmeans(model_val, specs = "age.terciles")
pairs(emm_val)
```

```
##  contrast       estimate    SE   df t.ratio p.value
##  Young - Middle  0.37990 0.121 1035   3.142  0.0049
##  Young - Old     0.37859 0.124 1035   3.041  0.0068
##  Middle - Old   -0.00131 0.123 1035  -0.011  0.9999
## 
## P value adjustment: tukey method for comparing a family of 3 estimates
```

# Section 4: Research Question 3 - Perception of the specific event types across age

```
vacation <- filter(data, event_type == "Vacation")
occasion <- filter(data, event_type == "Celebrating a special occasion")
illness <- filter(data, event_type == "Illness or injury")
death <- filter(data, event_type == "Death of a loved one")
```

## Age as predictor

### Table

```
## Vacation
gam_chall_vacation <- gam(ecq.chall ~ s(age), data = vacation, method = "REML")
res_chall_vacation <- c(round(summary(gam_chall_vacation)$edf,2), round(summary(gam_chall_vacation)$s.pv,3), 
               round(summary(gam_chall_vacation)$r.sq,3))

gam_changeww_vacation <- gam(ecq.changeww ~ s(age), data = vacation, method = "REML")
res_changeww_vacation <- c(round(summary(gam_changeww_vacation)$edf,2), round(summary(gam_changeww_vacation)$s.pv,3), 
               round(summary(gam_changeww_vacation)$r.sq,3))

gam_emosig_vacation <- gam(ecq.emosig ~ s(age), data = vacation, method = "REML")
res_emosig_vacation <- c(round(summary(gam_emosig_vacation)$edf,2), round(summary(gam_emosig_vacation)$s.pv,3), 
               round(summary(gam_emosig_vacation)$r.sq,3))

gam_extrao_vacation <- gam(ecq.extrao ~ s(age), data = vacation, method = "REML")
res_extrao_vacation <- c(round(summary(gam_extrao_vacation)$edf,2), round(summary(gam_extrao_vacation)$s.pv,3), 
               round(summary(gam_extrao_vacation)$r.sq,3))

gam_extcon_vacation <- gam(ecq.extcon ~ s(age), data = vacation, method = "REML")
res_extcon_vacation <- c(round(summary(gam_extcon_vacation)$edf,2), round(summary(gam_extcon_vacation)$s.pv,3), 
               round(summary(gam_extcon_vacation)$r.sq,3))

gam_impact_vacation <- gam(ecq.impact ~ s(age), data = vacation, method = "REML")
res_impact_vacation <- c(round(summary(gam_impact_vacation)$edf,2), round(summary(gam_impact_vacation)$s.pv,3), 
               round(summary(gam_impact_vacation)$r.sq,3))

gam_predict_vacation <- gam(ecq.predict ~ s(age), data = vacation, method = "REML")
res_predict_vacation <- c(round(summary(gam_predict_vacation)$edf,2), round(summary(gam_predict_vacation)$s.pv,3), 
               round(summary(gam_predict_vacation)$r.sq,3))

gam_socstat_vacation <- gam(ecq.socstat ~ s(age), data = vacation, method = "REML")
res_socstat_vacation <- c(round(summary(gam_socstat_vacation)$edf,2), round(summary(gam_socstat_vacation)$s.pv,3), 
               round(summary(gam_socstat_vacation)$r.sq,3))

gam_val_vacation <- gam(ecq.val ~ s(age), data = vacation, method = "REML")
res_val_vacation <- c(round(summary(gam_val_vacation)$edf,2), round(summary(gam_val_vacation)$s.pv,3), 
               round(summary(gam_val_vacation)$r.sq,3))

## Combine results
res_gam_vacation <- as.data.frame(rbind(res_chall_vacation, res_changeww_vacation, res_emosig_vacation,
                               res_extcon_vacation, res_extrao_vacation, res_impact_vacation,
                               res_predict_vacation, res_socstat_vacation, res_val_vacation))


## Celebrating a special occasion 
gam_chall_occasion <- gam(ecq.chall ~ s(age), data = occasion, method = "REML")
res_chall_occasion <- c(round(summary(gam_chall_occasion)$edf,2), round(summary(gam_chall_occasion)$s.pv,3), 
               round(summary(gam_chall_occasion)$r.sq,3))

gam_changeww_occasion <- gam(ecq.changeww ~ s(age), data = occasion, method = "REML")
res_changeww_occasion <- c(round(summary(gam_changeww_occasion)$edf,2), round(summary(gam_changeww_occasion)$s.pv,3), 
               round(summary(gam_changeww_occasion)$r.sq,3))

gam_emosig_occasion <- gam(ecq.emosig ~ s(age), data = occasion, method = "REML")
res_emosig_occasion <- c(round(summary(gam_emosig_occasion)$edf,2), round(summary(gam_emosig_occasion)$s.pv,3), 
               round(summary(gam_emosig_occasion)$r.sq,3))

gam_extrao_occasion <- gam(ecq.extrao ~ s(age), data = occasion, method = "REML")
res_extrao_occasion <- c(round(summary(gam_extrao_occasion)$edf,2), round(summary(gam_extrao_occasion)$s.pv,3), 
               round(summary(gam_extrao_occasion)$r.sq,3))

gam_extcon_occasion <- gam(ecq.extcon ~ s(age), data = occasion, method = "REML")
res_extcon_occasion <- c(round(summary(gam_extcon_occasion)$edf,2), round(summary(gam_extcon_occasion)$s.pv,3), 
               round(summary(gam_extcon_occasion)$r.sq,3))

gam_impact_occasion <- gam(ecq.impact ~ s(age), data = occasion, method = "REML")
res_impact_occasion <- c(round(summary(gam_impact_occasion)$edf,2), round(summary(gam_impact_occasion)$s.pv,3), 
               round(summary(gam_impact_occasion)$r.sq,3))

gam_predict_occasion <- gam(ecq.predict ~ s(age), data = occasion, method = "REML")
res_predict_occasion <- c(round(summary(gam_predict_occasion)$edf,2), round(summary(gam_predict_occasion)$s.pv,3), 
               round(summary(gam_predict_occasion)$r.sq,3))

gam_socstat_occasion <- gam(ecq.socstat ~ s(age), data = occasion, method = "REML")
res_socstat_occasion <- c(round(summary(gam_socstat_occasion)$edf,2), round(summary(gam_socstat_occasion)$s.pv,3), 
               round(summary(gam_socstat_occasion)$r.sq,3))

gam_val_occasion <- gam(ecq.val ~ s(age), data = occasion, method = "REML")
res_val_occasion <- c(round(summary(gam_val_occasion)$edf,2), round(summary(gam_val_occasion)$s.pv,3), 
               round(summary(gam_val_occasion)$r.sq,3))

## Combine results
res_gam_occasion <- as.data.frame(rbind(res_chall_occasion, res_changeww_occasion, res_emosig_occasion,
                               res_extcon_occasion, res_extrao_occasion, res_impact_occasion,
                               res_predict_occasion, res_socstat_occasion, res_val_occasion))


## Illness or injury
gam_chall_illness <- gam(ecq.chall ~ s(age), data = illness, method = "REML")
res_chall_illness <- c(round(summary(gam_chall_illness)$edf,2), round(summary(gam_chall_illness)$s.pv,3), 
               round(summary(gam_chall_illness)$r.sq,3))

gam_changeww_illness <- gam(ecq.changeww ~ s(age), data = illness, method = "REML")
res_changeww_illness <- c(round(summary(gam_changeww_illness)$edf,2), round(summary(gam_changeww_illness)$s.pv,3), 
               round(summary(gam_changeww_illness)$r.sq,3))

gam_emosig_illness <- gam(ecq.emosig ~ s(age), data = illness, method = "REML")
res_emosig_illness <- c(round(summary(gam_emosig_illness)$edf,2), round(summary(gam_emosig_illness)$s.pv,3), 
               round(summary(gam_emosig_illness)$r.sq,3))

gam_extrao_illness <- gam(ecq.extrao ~ s(age), data = illness, method = "REML")
res_extrao_illness <- c(round(summary(gam_extrao_illness)$edf,2), round(summary(gam_extrao_illness)$s.pv,3), 
               round(summary(gam_extrao_illness)$r.sq,3))

gam_extcon_illness <- gam(ecq.extcon ~ s(age), data = illness, method = "REML")
res_extcon_illness <- c(round(summary(gam_extcon_illness)$edf,2), round(summary(gam_extcon_illness)$s.pv,3), 
               round(summary(gam_extcon_illness)$r.sq,3))

gam_impact_illness <- gam(ecq.impact ~ s(age), data = illness, method = "REML")
res_impact_illness <- c(round(summary(gam_impact_illness)$edf,2), round(summary(gam_impact_illness)$s.pv,3), 
               round(summary(gam_impact_illness)$r.sq,3))

gam_predict_illness <- gam(ecq.predict ~ s(age), data = illness, method = "REML")
res_predict_illness <- c(round(summary(gam_predict_illness)$edf,2), round(summary(gam_predict_illness)$s.pv,3), 
               round(summary(gam_predict_illness)$r.sq,3))

gam_socstat_illness <- gam(ecq.socstat ~ s(age), data = illness, method = "REML")
res_socstat_illness <- c(round(summary(gam_socstat_illness)$edf,2), round(summary(gam_socstat_illness)$s.pv,3), 
               round(summary(gam_socstat_illness)$r.sq,3))

gam_val_illness <- gam(ecq.val ~ s(age), data = illness, method = "REML")
res_val_illness <- c(round(summary(gam_val_illness)$edf,2), round(summary(gam_val_illness)$s.pv,3), 
               round(summary(gam_val_illness)$r.sq,3))

## Combine results
res_gam_illness <- as.data.frame(rbind(res_chall_illness, res_changeww_illness, res_emosig_illness,
                               res_extcon_illness, res_extrao_illness, res_impact_illness,
                               res_predict_illness, res_socstat_illness, res_val_illness))


## Death of a loved one
gam_chall_death <- gam(ecq.chall ~ s(age), data = death, method = "REML")
res_chall_death <- c(round(summary(gam_chall_death)$edf,2), round(summary(gam_chall_death)$s.pv,3), 
               round(summary(gam_chall_death)$r.sq,3))

gam_changeww_death <- gam(ecq.changeww ~ s(age), data = death, method = "REML")
res_changeww_death <- c(round(summary(gam_changeww_death)$edf,2), round(summary(gam_changeww_death)$s.pv,3), 
               round(summary(gam_changeww_death)$r.sq,3))

gam_emosig_death <- gam(ecq.emosig ~ s(age), data = death, method = "REML")
res_emosig_death <- c(round(summary(gam_emosig_death)$edf,2), round(summary(gam_emosig_death)$s.pv,3), 
               round(summary(gam_emosig_death)$r.sq,3))

gam_extrao_death <- gam(ecq.extrao ~ s(age), data = death, method = "REML")
res_extrao_death <- c(round(summary(gam_extrao_death)$edf,2), round(summary(gam_extrao_death)$s.pv,3), 
               round(summary(gam_extrao_death)$r.sq,3))

gam_extcon_death <- gam(ecq.extcon ~ s(age), data = death, method = "REML")
res_extcon_death <- c(round(summary(gam_extcon_death)$edf,2), round(summary(gam_extcon_death)$s.pv,3), 
               round(summary(gam_extcon_death)$r.sq,3))

gam_impact_death <- gam(ecq.impact ~ s(age), data = death, method = "REML")
res_impact_death <- c(round(summary(gam_impact_death)$edf,2), round(summary(gam_impact_death)$s.pv,3), 
               round(summary(gam_impact_death)$r.sq,3))

gam_predict_death <- gam(ecq.predict ~ s(age), data = death, method = "REML")
res_predict_death <- c(round(summary(gam_predict_death)$edf,2), round(summary(gam_predict_death)$s.pv,3), 
               round(summary(gam_predict_death)$r.sq,3))

gam_socstat_death <- gam(ecq.socstat ~ s(age), data = death, method = "REML")
res_socstat_death <- c(round(summary(gam_socstat_death)$edf,2), round(summary(gam_socstat_death)$s.pv,3), 
               round(summary(gam_socstat_death)$r.sq,3))

gam_val_death <- gam(ecq.val ~ s(age), data = death, method = "REML")
res_val_death <- c(round(summary(gam_val_death)$edf,2), round(summary(gam_val_death)$s.pv,3), 
               round(summary(gam_val_death)$r.sq,3))

## Combine results
res_gam_death <- as.data.frame(rbind(res_chall_death, res_changeww_death, res_emosig_death,
                               res_extcon_death, res_extrao_death, res_impact_death,
                               res_predict_death, res_socstat_death, res_val_death))


## Create table
res_events_age <- cbind(res_gam_vacation, res_gam_occasion, 
                        res_gam_illness,  res_gam_death)

names(res_events_age) <- rep(c("Edf", "p", "R^2^"),4)
row.names(res_events_age) <- c("Challenge", "Change in world views", 
                        "Emotional significance", "External control", 
                        "Extraordinariness", "Impact",
                        "Predictability", "Social status change", 
                        "Valence")
kable(res_events_age
      , caption="Results of GAMs with a smoothed age-term as predictor"
      , escape=FALSE
      , label = NA) %>% 
  kable_styling(bootstrap_options = c("striped", "hover"), 
                fixed_thead = T, full_width = FALSE, position="left") %>% 
  add_header_above(c(" " = 1, "Vacation" = 3, "Celebrating a special occasion" = 3, 
                   "Illness or injury" = 3, 
                   "Death of a loved one" = 3))
```

Results of GAMs with a smoothed age-term as predictor

|  | Vacation | | | Celebrating a special occasion | | | Illness or injury | | | Death of a loved one | | |
| --- | --- | --- | --- | --- | --- | --- | --- | --- | --- | --- | --- | --- |
|  | Edf | p | R2 | Edf | p | R2 | Edf | p | R2 | Edf | p | R2 |
| Challenge | 2.25 | 0.033 | 0.061 | 1.12 | 0.079 | 0.032 | 1.00 | 0.420 | -0.005 | 2.12 | 0.250 | 0.040 |
| Change in world views | 1.00 | 0.962 | -0.008 | 1.00 | 0.154 | 0.012 | 2.71 | 0.026 | 0.119 | 2.22 | 0.148 | 0.060 |
| Emotional significance | 1.00 | 0.781 | -0.007 | 1.00 | 0.391 | -0.003 | 1.00 | 0.457 | -0.006 | 1.00 | 0.681 | -0.012 |
| External control | 2.35 | 0.163 | 0.034 | 1.95 | 0.554 | 0.013 | 1.00 | 0.406 | -0.004 | 1.00 | 0.919 | -0.015 |
| Extraordinariness | 3.40 | 0.038 | 0.084 | 1.00 | 0.424 | -0.005 | 1.00 | 0.672 | -0.011 | 1.00 | 0.245 | 0.006 |
| Impact | 1.00 | 0.633 | -0.006 | 2.75 | 0.121 | 0.067 | 1.00 | 0.362 | -0.002 | 2.29 | 0.184 | 0.054 |
| Predictability | 2.52 | 0.023 | 0.072 | 2.14 | 0.490 | 0.019 | 1.00 | 0.350 | -0.002 | 1.00 | 0.007 | 0.089 |
| Social status change | 1.00 | 0.234 | 0.003 | 2.27 | 0.076 | 0.070 | 2.29 | 0.217 | 0.048 | 1.57 | 0.572 | 0.003 |
| Valence | 1.00 | 0.939 | -0.008 | 6.08 | 0.001 | 0.255 | 1.00 | 0.466 | -0.006 | 1.52 | 0.307 | 0.021 |

### Graph

```
## Challenge
res_chall_vacation <- predict_gam(gam_chall_vacation, length_out = 1000) 
res_chall_vacation$event <- "Vacation"
res_chall_occasion <- predict_gam(gam_chall_occasion, length_out = 1000)
res_chall_occasion$event <- "Special occasion"
res_chall_illness <- predict_gam(gam_chall_illness, length_out = 1000)
res_chall_illness$event <- "Illness or injury"
res_chall_death<- predict_gam(gam_chall_death, length_out = 1000)
res_chall_death$event <- "Death of a loved one"

res_chall_graph <- rbind(res_chall_vacation, 
                         res_chall_occasion, 
                         res_chall_illness, 
                         res_chall_death)

plot_chall_age <- ggplot(aes(x = age, y = ecq.chall), data = res_chall_graph) +
  geom_line(aes(by = event, colour = event), size = 1) +
  scale_y_continuous(limits = c(0,6)) +
  geom_ribbon(aes(ymin = lower_ci, ymax = upper_ci, by = event, fill = event), alpha = 0.20) +
  theme_apa() +
    scale_color_manual(values = c(1,2,3,4,5)) +
  scale_fill_manual(values = c(1,2,3,4,5)) +
  labs(x = "Age", y = "Challenge") +
  theme(legend.position = "none",
        legend.text = element_text(size = 14), 
        axis.title = element_text(size = 14), 
        axis.text = element_text(size = 14), 
        legend.title = element_text(size = 14))


## Change in world views
res_changeww_vacation <- predict_gam(gam_changeww_vacation, length_out = 1000) 
res_changeww_vacation$event <- "Vacation"
res_changeww_occasion <- predict_gam(gam_changeww_occasion, length_out = 1000)
res_changeww_occasion$event <- "Special occasion"
res_changeww_illness <- predict_gam(gam_changeww_illness, length_out = 1000)
res_changeww_illness$event <- "Illness or injury"
res_changeww_death<- predict_gam(gam_changeww_death, length_out = 1000)
res_changeww_death$event <- "Death of a loved one"

res_changeww_graph <- rbind(res_changeww_vacation, 
                         res_changeww_occasion, 
                         res_changeww_illness, 
                         res_changeww_death)

plot_changeww_age <- ggplot(aes(x = age, y = ecq.changeww), data = res_changeww_graph) +
  geom_line(aes(by = event, colour = event), size = 1) +
  scale_y_continuous(limits = c(0,6)) +
  geom_ribbon(aes(ymin = lower_ci, ymax = upper_ci, by = event, fill = event), alpha = 0.20) +
  theme_apa() +
    scale_color_manual(values = c(1,2,3,4,5)) +
  scale_fill_manual(values = c(1,2,3,4,5)) +
  labs(x = "Age", y = "Change in world views") +
  theme(legend.position = "none",
        legend.text = element_text(size = 14), 
        axis.title = element_text(size = 14), 
        axis.text = element_text(size = 14), 
        legend.title = element_text(size = 14))


## Emotional significance
res_emosig_vacation <- predict_gam(gam_emosig_vacation, length_out = 1000) 
res_emosig_vacation$event <- "Vacation"
res_emosig_occasion <- predict_gam(gam_emosig_occasion, length_out = 1000)
res_emosig_occasion$event <- "Special occasion"
res_emosig_illness <- predict_gam(gam_emosig_illness, length_out = 1000)
res_emosig_illness$event <- "Illness or injury"
res_emosig_death<- predict_gam(gam_emosig_death, length_out = 1000)
res_emosig_death$event <- "Death of a loved one"

res_emosig_graph <- rbind(res_emosig_vacation, 
                         res_emosig_occasion, 
                         res_emosig_illness, 
                         res_emosig_death)

plot_emosig_age <- ggplot(aes(x = age, y = ecq.emosig), data = res_emosig_graph) +
  geom_line(aes(by = event, colour = event), size = 1) +
  scale_y_continuous(limits = c(0,6)) +
  geom_ribbon(aes(ymin = lower_ci, ymax = upper_ci, by = event, fill = event), alpha = 0.20) +
  theme_apa() +
    scale_color_manual(values = c(1,2,3,4,5)) +
  scale_fill_manual(values = c(1,2,3,4,5)) +
  labs(x = "Age", y = "Emotional significance") +
  theme(legend.position = "none",
    legend.text = element_text(size = 14), 
        axis.title = element_text(size = 14), 
        axis.text = element_text(size = 14), 
        legend.title = element_text(size = 14))


## External control
res_extcon_vacation <- predict_gam(gam_extcon_vacation, length_out = 1000) 
res_extcon_vacation$event <- "Vacation"
res_extcon_occasion <- predict_gam(gam_extcon_occasion, length_out = 1000)
res_extcon_occasion$event <- "Special occasion"
res_extcon_illness <- predict_gam(gam_extcon_illness, length_out = 1000)
res_extcon_illness$event <- "Illness or injury"
res_extcon_death<- predict_gam(gam_extcon_death, length_out = 1000)
res_extcon_death$event <- "Death of a loved one"

res_extcon_graph <- rbind(res_extcon_vacation, 
                         res_extcon_occasion, 
                         res_extcon_illness, 
                         res_extcon_death)

plot_extcon_age <- ggplot(aes(x = age, y = ecq.extcon), data = res_extcon_graph) +
  geom_line(aes(by = event, colour = event), size = 1) +
  scale_y_continuous(limits = c(0,6)) +
  geom_ribbon(aes(ymin = lower_ci, ymax = upper_ci, by = event, fill = event), alpha = 0.20) +
  theme_apa() +
    scale_color_manual(values = c(1,2,3,4,5)) +
  scale_fill_manual(values = c(1,2,3,4,5)) +
  labs(x = "Age", y = "External control") +
  theme(legend.position = "none",
    legend.text = element_text(size = 14), 
        axis.title = element_text(size = 14), 
        axis.text = element_text(size = 14), 
        legend.title = element_text(size = 14))


## Extraordinariness
res_extrao_vacation <- predict_gam(gam_extrao_vacation, length_out = 1000) 
res_extrao_vacation$event <- "Vacation"
res_extrao_occasion <- predict_gam(gam_extrao_occasion, length_out = 1000)
res_extrao_occasion$event <- "Special occasion"
res_extrao_illness <- predict_gam(gam_extrao_illness, length_out = 1000)
res_extrao_illness$event <- "Illness or injury"
res_extrao_death<- predict_gam(gam_extrao_death, length_out = 1000)
res_extrao_death$event <- "Death of a loved one"

res_extrao_graph <- rbind(res_extrao_vacation, 
                         res_extrao_occasion, 
                         res_extrao_illness, 
                         res_extrao_death)

plot_extrao_age <- ggplot(aes(x = age, y = ecq.extrao), data = res_extrao_graph) +
  geom_line(aes(by = event, colour = event), size = 1) +
  scale_y_continuous(limits = c(0,6)) +
  geom_ribbon(aes(ymin = lower_ci, ymax = upper_ci, by = event, fill = event), alpha = 0.20) +
  theme_apa() +
    scale_color_manual(values = c(1,2,3,4,5)) +
  scale_fill_manual(values = c(1,2,3,4,5)) +
  labs(x = "Age", y = "Extraordinariness") +
  theme(legend.position = "none",
    legend.text = element_text(size = 14), 
        axis.title = element_text(size = 14), 
        axis.text = element_text(size = 14), 
        legend.title = element_text(size = 14))


## Impact
res_impact_vacation <- predict_gam(gam_impact_vacation, length_out = 1000) 
res_impact_vacation$event <- "Vacation"
res_impact_occasion <- predict_gam(gam_impact_occasion, length_out = 1000)
res_impact_occasion$event <- "Special occasion"
res_impact_illness <- predict_gam(gam_impact_illness, length_out = 1000)
res_impact_illness$event <- "Illness or injury"
res_impact_death<- predict_gam(gam_impact_death, length_out = 1000)
res_impact_death$event <- "Death of a loved one"

res_impact_graph <- rbind(res_impact_vacation, 
                         res_impact_occasion, 
                         res_impact_illness, 
                         res_impact_death)

plot_impact_age <- ggplot(aes(x = age, y = ecq.impact), data = res_impact_graph) +
  geom_line(aes(by = event, colour = event), size = 1) +
  scale_y_continuous(limits = c(0,6)) +
  scale_color_manual(values = c(1,2,3,4,5)) +
  scale_fill_manual(values = c(1,2,3,4,5)) +
  geom_ribbon(aes(ymin = lower_ci, ymax = upper_ci, by = event, fill = event), alpha = 0.20) +
  theme_apa() +
    scale_color_manual(values = c(1,2,3,4,5)) +
  scale_fill_manual(values = c(1,2,3,4,5)) +
  labs(x = "Age", y = "Impact") +
  theme(legend.position = "none",
    legend.text = element_text(size = 14), 
        axis.title = element_text(size = 14), 
        axis.text = element_text(size = 14), 
        legend.title = element_text(size = 14))


## Predictability
res_predict_vacation <- predict_gam(gam_predict_vacation, length_out = 1000) 
res_predict_vacation$event <- "Vacation"
res_predict_occasion <- predict_gam(gam_predict_occasion, length_out = 1000)
res_predict_occasion$event <- "Special occasion"
res_predict_illness <- predict_gam(gam_predict_illness, length_out = 1000)
res_predict_illness$event <- "Illness or injury"
res_predict_death<- predict_gam(gam_predict_death, length_out = 1000)
res_predict_death$event <- "Death of a loved one"

res_predict_graph <- rbind(res_predict_vacation, 
                         res_predict_occasion, 
                         res_predict_illness, 
                         res_predict_death)

plot_predict_age <- ggplot(aes(x = age, y = ecq.predict), data = res_predict_graph) +
  geom_line(aes(by = event, colour = event), size = 1) +
  scale_y_continuous(limits = c(0,6)) +
  geom_ribbon(aes(ymin = lower_ci, ymax = upper_ci, by = event, fill = event), alpha = 0.20) +
  scale_color_manual(values = c(1,2,3,4,5)) +
  scale_fill_manual(values = c(1,2,3,4,5)) +
  theme_apa() +
  labs(x = "Age", y = "Predictability") +
  theme(legend.position = "none",
    legend.text = element_text(size = 14), 
        axis.title = element_text(size = 14), 
        axis.text = element_text(size = 14), 
        legend.title = element_text(size = 14))


## Social status change
res_socstat_vacation <- predict_gam(gam_socstat_vacation, length_out = 1000) 
res_socstat_vacation$event <- "Vacation"
res_socstat_occasion <- predict_gam(gam_socstat_occasion, length_out = 1000)
res_socstat_occasion$event <- "Special occasion"
res_socstat_illness <- predict_gam(gam_socstat_illness, length_out = 1000)
res_socstat_illness$event <- "Illness or injury"
res_socstat_death<- predict_gam(gam_socstat_death, length_out = 1000)
res_socstat_death$event <- "Death of a loved one"

res_socstat_graph <- rbind(res_socstat_vacation, 
                         res_socstat_occasion, 
                         res_socstat_illness, 
                         res_socstat_death)

plot_socstat_age <- ggplot(aes(x = age, y = ecq.socstat), data = res_socstat_graph) +
  geom_line(aes(by = event, colour = event), size = 1) +
  scale_y_continuous(limits = c(0,6)) +
    scale_color_manual(values = c(1,2,3,4,5)) +
  scale_fill_manual(values = c(1,2,3,4,5)) +
  geom_ribbon(aes(ymin = lower_ci, ymax = upper_ci, by = event, fill = event), alpha = 0.20) +
  theme_apa() +
  labs(x = "Age", y = "Social status change") +
  theme(legend.position = "none",
        legend.text = element_text(size = 14), 
        axis.title = element_text(size = 14), 
        axis.text = element_text(size = 14), 
        legend.title = element_text(size = 14))


## Valence
res_val_vacation <- predict_gam(gam_val_vacation, length_out = 1000) 
res_val_vacation$event <- "Vacation"
res_val_occasion <- predict_gam(gam_val_occasion, length_out = 1000)
res_val_occasion$event <- "Special occasion"
res_val_illness <- predict_gam(gam_val_illness, length_out = 1000)
res_val_illness$event <- "Illness or injury"
res_val_death<- predict_gam(gam_val_death, length_out = 1000)
res_val_death$event <- "Death of a loved one"

res_val_graph <- rbind(res_val_vacation, 
                       res_val_occasion, 
                       res_val_illness, 
                       res_val_death)

plot_val_age <- ggplot(aes(x = age, y = ecq.val), data = res_val_graph) +
  geom_line(aes(by = event, colour = event), size = 1) +
  scale_y_continuous(limits = c(0,6)) +
    scale_color_manual(values = c(1,2,3,4,5)) +
  scale_fill_manual(values = c(1,2,3,4,5)) +
  geom_ribbon(aes(ymin = lower_ci, ymax = upper_ci, by = event, fill = event), alpha = 0.20) +
  theme_apa() +
  labs(x = "Age", y = "Valence", color = "Event", fill = "Event") +
  theme(legend.position = "none",
    legend.text = element_text(size = 14), 
        axis.title = element_text(size = 14), 
        axis.text = element_text(size = 14), 
        legend.title = element_text(size = 14))

plot_val_age2 <- ggplot(aes(x = age, y = ecq.val), data = res_val_graph) +
  geom_line(aes(by = event, colour = event), size = 1) +
  scale_y_continuous(limits = c(0,6)) +
    scale_color_manual(values = c(1,2,3,4,5)) +
  scale_fill_manual(values = c(1,2,3,4,5)) +
  geom_ribbon(aes(ymin = lower_ci, ymax = upper_ci, by = event, fill = event), alpha = 0.20) +
  theme_apa() +
  labs(x = "Age", y = "Valence", color = "Event", fill = "Event") +
  theme(legend.position = "right",
    legend.text = element_text(size = 14), 
        axis.title = element_text(size = 14), 
        axis.text = element_text(size = 14), 
        legend.title = element_text(size = 14))

legend <- cowplot::get_legend(plot_val_age2)

cowplot::plot_grid(plot_chall_age, plot_changeww_age, plot_emosig_age, 
                   plot_extcon_age, plot_extrao_age, plot_impact_age, 
                   plot_predict_age, plot_socstat_age, plot_val_age, 
                   NULL, legend, NULL, ncol = 3, nrow = 4, 
                   rel_heights = c(1,1,1,0.5))
```

```
## ggsave("PLot4.svg", height = 13, width = 10)
```

## Age-normativity as predictor

### Normative age

```
## compute age normativity predictor
vacation$normativity <- abs(vacation$age - mean(vacation$age, na.rm = TRUE))
occasion$normativity <- abs(occasion$age - mean(occasion$age, na.rm = TRUE))
illness$normativity <- abs(illness$age - mean(illness$age, na.rm = TRUE))
death$normativity <- abs(death$age - mean(death$age, na.rm = TRUE))
```

Vacation: The event is on average experienced at 57.46 years (SD = 15.81).

Celebrating a special occasion: The event is on average experienced at 59.59 years (SD = 17.93).

Illness or injury: The event is on average experienced at 61.93 years (SD = 16.15).

Death of a loved one: The event is on average experienced at 59.34 years (SD = 14.87).

### Table

```
## Vacation
gam_chall_vacation <- gam(ecq.chall ~ s(normativity), data = vacation, method = "REML")
res_chall_vacation <- c(round(summary(gam_chall_vacation)$edf,2), round(summary(gam_chall_vacation)$s.pv,3), 
               round(summary(gam_chall_vacation)$r.sq,3))

gam_changeww_vacation <- gam(ecq.changeww ~ s(normativity), data = vacation, method = "REML")
res_changeww_vacation <- c(round(summary(gam_changeww_vacation)$edf,2), round(summary(gam_changeww_vacation)$s.pv,3), 
               round(summary(gam_changeww_vacation)$r.sq,3))

gam_emosig_vacation <- gam(ecq.emosig ~ s(normativity), data = vacation, method = "REML")
res_emosig_vacation <- c(round(summary(gam_emosig_vacation)$edf,2), round(summary(gam_emosig_vacation)$s.pv,3), 
               round(summary(gam_emosig_vacation)$r.sq,3))

gam_extrao_vacation <- gam(ecq.extrao ~ s(normativity), data = vacation, method = "REML")
res_extrao_vacation <- c(round(summary(gam_extrao_vacation)$edf,2), round(summary(gam_extrao_vacation)$s.pv,3), 
               round(summary(gam_extrao_vacation)$r.sq,3))

gam_extcon_vacation <- gam(ecq.extcon ~ s(normativity), data = vacation, method = "REML")
res_extcon_vacation <- c(round(summary(gam_extcon_vacation)$edf,2), round(summary(gam_extcon_vacation)$s.pv,3), 
               round(summary(gam_extcon_vacation)$r.sq,3))

gam_impact_vacation <- gam(ecq.impact ~ s(normativity), data = vacation, method = "REML")
res_impact_vacation <- c(round(summary(gam_impact_vacation)$edf,2), round(summary(gam_impact_vacation)$s.pv,3), 
               round(summary(gam_impact_vacation)$r.sq,3))

gam_predict_vacation <- gam(ecq.predict ~ s(normativity), data = vacation, method = "REML")
res_predict_vacation <- c(round(summary(gam_predict_vacation)$edf,2), round(summary(gam_predict_vacation)$s.pv,3), 
               round(summary(gam_predict_vacation)$r.sq,3))

gam_socstat_vacation <- gam(ecq.socstat ~ s(normativity), data = vacation, method = "REML")
res_socstat_vacation <- c(round(summary(gam_socstat_vacation)$edf,2), round(summary(gam_socstat_vacation)$s.pv,3), 
               round(summary(gam_socstat_vacation)$r.sq,3))

gam_val_vacation <- gam(ecq.val ~ s(normativity), data = vacation, method = "REML")
res_val_vacation <- c(round(summary(gam_val_vacation)$edf,2), round(summary(gam_val_vacation)$s.pv,3), 
               round(summary(gam_val_vacation)$r.sq,3))

## Combine results
res_gam_vacation <- as.data.frame(rbind(res_chall_vacation, res_changeww_vacation, res_emosig_vacation,
                               res_extcon_vacation, res_extrao_vacation, res_impact_vacation,
                               res_predict_vacation, res_socstat_vacation, res_val_vacation))


## Celebrating a special occasion 
gam_chall_occasion <- gam(ecq.chall ~ s(normativity), data = occasion, method = "REML")
res_chall_occasion <- c(round(summary(gam_chall_occasion)$edf,2), round(summary(gam_chall_occasion)$s.pv,3), 
               round(summary(gam_chall_occasion)$r.sq,3))

gam_changeww_occasion <- gam(ecq.changeww ~ s(normativity), data = occasion, method = "REML")
res_changeww_occasion <- c(round(summary(gam_changeww_occasion)$edf,2), round(summary(gam_changeww_occasion)$s.pv,3), 
               round(summary(gam_changeww_occasion)$r.sq,3))

gam_emosig_occasion <- gam(ecq.emosig ~ s(normativity), data = occasion, method = "REML")
res_emosig_occasion <- c(round(summary(gam_emosig_occasion)$edf,2), round(summary(gam_emosig_occasion)$s.pv,3), 
               round(summary(gam_emosig_occasion)$r.sq,3))

gam_extrao_occasion <- gam(ecq.extrao ~ s(normativity), data = occasion, method = "REML")
res_extrao_occasion <- c(round(summary(gam_extrao_occasion)$edf,2), round(summary(gam_extrao_occasion)$s.pv,3), 
               round(summary(gam_extrao_occasion)$r.sq,3))

gam_extcon_occasion <- gam(ecq.extcon ~ s(normativity), data = occasion, method = "REML")
res_extcon_occasion <- c(round(summary(gam_extcon_occasion)$edf,2), round(summary(gam_extcon_occasion)$s.pv,3), 
               round(summary(gam_extcon_occasion)$r.sq,3))

gam_impact_occasion <- gam(ecq.impact ~ s(normativity), data = occasion, method = "REML")
res_impact_occasion <- c(round(summary(gam_impact_occasion)$edf,2), round(summary(gam_impact_occasion)$s.pv,3), 
               round(summary(gam_impact_occasion)$r.sq,3))

gam_predict_occasion <- gam(ecq.predict ~ s(normativity), data = occasion, method = "REML")
res_predict_occasion <- c(round(summary(gam_predict_occasion)$edf,2), round(summary(gam_predict_occasion)$s.pv,3), 
               round(summary(gam_predict_occasion)$r.sq,3))

gam_socstat_occasion <- gam(ecq.socstat ~ s(normativity), data = occasion, method = "REML")
res_socstat_occasion <- c(round(summary(gam_socstat_occasion)$edf,2), round(summary(gam_socstat_occasion)$s.pv,3), 
               round(summary(gam_socstat_occasion)$r.sq,3))

gam_val_occasion <- gam(ecq.val ~ s(normativity), data = occasion, method = "REML")
res_val_occasion <- c(round(summary(gam_val_occasion)$edf,2), round(summary(gam_val_occasion)$s.pv,3), 
               round(summary(gam_val_occasion)$r.sq,3))

## Combine results
res_gam_occasion <- as.data.frame(rbind(res_chall_occasion, res_changeww_occasion, res_emosig_occasion,
                               res_extcon_occasion, res_extrao_occasion, res_impact_occasion,
                               res_predict_occasion, res_socstat_occasion, res_val_occasion))


## Illness or injury
gam_chall_illness <- gam(ecq.chall ~ s(normativity), data = illness, method = "REML")
res_chall_illness <- c(round(summary(gam_chall_illness)$edf,2), round(summary(gam_chall_illness)$s.pv,3), 
               round(summary(gam_chall_illness)$r.sq,3))

gam_changeww_illness <- gam(ecq.changeww ~ s(normativity), data = illness, method = "REML")
res_changeww_illness <- c(round(summary(gam_changeww_illness)$edf,2), round(summary(gam_changeww_illness)$s.pv,3), 
               round(summary(gam_changeww_illness)$r.sq,3))

gam_emosig_illness <- gam(ecq.emosig ~ s(normativity), data = illness, method = "REML")
res_emosig_illness <- c(round(summary(gam_emosig_illness)$edf,2), round(summary(gam_emosig_illness)$s.pv,3), 
               round(summary(gam_emosig_illness)$r.sq,3))

gam_extrao_illness <- gam(ecq.extrao ~ s(normativity), data = illness, method = "REML")
res_extrao_illness <- c(round(summary(gam_extrao_illness)$edf,2), round(summary(gam_extrao_illness)$s.pv,3), 
               round(summary(gam_extrao_illness)$r.sq,3))

gam_extcon_illness <- gam(ecq.extcon ~ s(normativity), data = illness, method = "REML")
res_extcon_illness <- c(round(summary(gam_extcon_illness)$edf,2), round(summary(gam_extcon_illness)$s.pv,3), 
               round(summary(gam_extcon_illness)$r.sq,3))

gam_impact_illness <- gam(ecq.impact ~ s(normativity), data = illness, method = "REML")
res_impact_illness <- c(round(summary(gam_impact_illness)$edf,2), round(summary(gam_impact_illness)$s.pv,3), 
               round(summary(gam_impact_illness)$r.sq,3))

gam_predict_illness <- gam(ecq.predict ~ s(normativity), data = illness, method = "REML")
res_predict_illness <- c(round(summary(gam_predict_illness)$edf,2), round(summary(gam_predict_illness)$s.pv,3), 
               round(summary(gam_predict_illness)$r.sq,3))

gam_socstat_illness <- gam(ecq.socstat ~ s(normativity), data = illness, method = "REML")
res_socstat_illness <- c(round(summary(gam_socstat_illness)$edf,2), round(summary(gam_socstat_illness)$s.pv,3), 
               round(summary(gam_socstat_illness)$r.sq,3))

gam_val_illness <- gam(ecq.val ~ s(normativity), data = illness, method = "REML")
res_val_illness <- c(round(summary(gam_val_illness)$edf,2), round(summary(gam_val_illness)$s.pv,3), 
               round(summary(gam_val_illness)$r.sq,3))

## Combine results
res_gam_illness <- as.data.frame(rbind(res_chall_illness, res_changeww_illness, res_emosig_illness,
                               res_extcon_illness, res_extrao_illness, res_impact_illness,
                               res_predict_illness, res_socstat_illness, res_val_illness))


## Death of a loved one
gam_chall_death <- gam(ecq.chall ~ s(normativity), data = death, method = "REML")
res_chall_death <- c(round(summary(gam_chall_death)$edf,2), round(summary(gam_chall_death)$s.pv,3), 
               round(summary(gam_chall_death)$r.sq,3))

gam_changeww_death <- gam(ecq.changeww ~ s(normativity), data = death, method = "REML")
res_changeww_death <- c(round(summary(gam_changeww_death)$edf,2), round(summary(gam_changeww_death)$s.pv,3), 
               round(summary(gam_changeww_death)$r.sq,3))

gam_emosig_death <- gam(ecq.emosig ~ s(normativity), data = death, method = "REML")
res_emosig_death <- c(round(summary(gam_emosig_death)$edf,2), round(summary(gam_emosig_death)$s.pv,3), 
               round(summary(gam_emosig_death)$r.sq,3))

gam_extrao_death <- gam(ecq.extrao ~ s(normativity), data = death, method = "REML")
res_extrao_death <- c(round(summary(gam_extrao_death)$edf,2), round(summary(gam_extrao_death)$s.pv,3), 
               round(summary(gam_extrao_death)$r.sq,3))

gam_extcon_death <- gam(ecq.extcon ~ s(normativity), data = death, method = "REML")
res_extcon_death <- c(round(summary(gam_extcon_death)$edf,2), round(summary(gam_extcon_death)$s.pv,3), 
               round(summary(gam_extcon_death)$r.sq,3))

gam_impact_death <- gam(ecq.impact ~ s(normativity), data = death, method = "REML")
res_impact_death <- c(round(summary(gam_impact_death)$edf,2), round(summary(gam_impact_death)$s.pv,3), 
               round(summary(gam_impact_death)$r.sq,3))

gam_predict_death <- gam(ecq.predict ~ s(normativity), data = death, method = "REML")
res_predict_death <- c(round(summary(gam_predict_death)$edf,2), round(summary(gam_predict_death)$s.pv,3), 
               round(summary(gam_predict_death)$r.sq,3))

gam_socstat_death <- gam(ecq.socstat ~ s(normativity), data = death, method = "REML")
res_socstat_death <- c(round(summary(gam_socstat_death)$edf,2), round(summary(gam_socstat_death)$s.pv,3), 
               round(summary(gam_socstat_death)$r.sq,3))

gam_val_death <- gam(ecq.val ~ s(normativity), data = death, method = "REML")
res_val_death <- c(round(summary(gam_val_death)$edf,2), round(summary(gam_val_death)$s.pv,3), 
               round(summary(gam_val_death)$r.sq,3))

## Combine results
res_gam_death <- as.data.frame(rbind(res_chall_death, res_changeww_death, res_emosig_death,
                               res_extcon_death, res_extrao_death, res_impact_death,
                               res_predict_death, res_socstat_death, res_val_death))


## Create table
res_events_normativity <- cbind(res_gam_vacation, res_gam_occasion, 
                        res_gam_illness,
                        res_gam_death)

names(res_events_normativity) <- rep(c("Edf", "p", "R^2^"),4)
row.names(res_events_normativity) <- c("Challenge", "Change in world views", 
                        "Emotional significance", "External control", 
                        "Extraordinariness", "Impact",
                        "Predictability", "Social status change", 
                        "Valence")
kable(res_events_normativity
      , caption="Results of GAMs with a smoothed normativity-term as predictor"
      , escape=FALSE
      , label = NA) %>% 
  kable_styling(bootstrap_options = c("striped", "hover"), 
                fixed_thead = T, full_width = FALSE, position="left") %>% 
  add_header_above(c(" " = 1, "Vacation" = 3, "Celebrating a special occasion" = 3, 
                   "Illness or injury" = 3, 
                   "Death of a loved one" = 3))
```

Results of GAMs with a smoothed normativity-term as predictor

|  | Vacation | | | Celebrating a special occasion | | | Illness or injury | | | Death of a loved one | | |
| --- | --- | --- | --- | --- | --- | --- | --- | --- | --- | --- | --- | --- |
|  | Edf | p | R2 | Edf | p | R2 | Edf | p | R2 | Edf | p | R2 |
| Challenge | 2.31 | 0.034 | 0.060 | 1.00 | 0.118 | 0.017 | 1.00 | 0.506 | -0.008 | 1.00 | 0.043 | 0.045 |
| Change in world views | 1.00 | 0.685 | -0.007 | 1.00 | 0.885 | -0.012 | 2.50 | 0.137 | 0.065 | 1.69 | 0.093 | 0.058 |
| Emotional significance | 1.00 | 0.892 | -0.008 | 1.00 | 0.912 | -0.012 | 1.71 | 0.538 | 0.005 | 1.00 | 0.398 | -0.004 |
| External control | 1.00 | 0.040 | 0.026 | 1.00 | 0.230 | 0.006 | 1.00 | 0.192 | 0.010 | 1.37 | 0.875 | -0.008 |
| Extraordinariness | 2.38 | 0.075 | 0.051 | 1.00 | 0.892 | -0.013 | 2.46 | 0.043 | 0.099 | 1.00 | 0.375 | -0.003 |
| Impact | 2.32 | 0.176 | 0.032 | 1.15 | 0.154 | 0.019 | 1.00 | 0.362 | -0.002 | 1.00 | 0.028 | 0.056 |
| Predictability | 1.01 | 0.056 | 0.022 | 1.00 | 0.127 | 0.016 | 1.88 | 0.447 | 0.014 | 1.00 | 0.632 | -0.011 |
| Social status change | 1.00 | 0.703 | -0.007 | 1.00 | 0.266 | 0.003 | 1.00 | 0.048 | 0.041 | 1.00 | 0.157 | 0.015 |
| Valence | 1.00 | 0.592 | -0.006 | 1.00 | 0.138 | 0.014 | 1.53 | 0.651 | -0.001 | 1.00 | 0.110 | 0.023 |

### Graph

```
## Challenge
res_chall_vacation <- predict_gam(gam_chall_vacation, length_out = 1000) 
res_chall_vacation$event <- "Vacation"
res_chall_occasion <- predict_gam(gam_chall_occasion, length_out = 1000)
res_chall_occasion$event <- "Special occasion"
res_chall_illness <- predict_gam(gam_chall_illness, length_out = 1000)
res_chall_illness$event <- "Illness or injury"
res_chall_death<- predict_gam(gam_chall_death, length_out = 1000)
res_chall_death$event <- "Death of a loved one"

res_chall_graph <- rbind(res_chall_vacation, 
                         res_chall_occasion, 
                         res_chall_illness, 
                         res_chall_death)

plot_chall_normativity <- ggplot(aes(x = normativity, y = ecq.chall), data = res_chall_graph) +
  geom_line(aes(by = event, colour = event), size = 1.3) +
  scale_y_continuous(limits = c(0,6)) +
  scale_color_manual(values = c(1,2,3,4,5)) +
  scale_fill_manual(values = c(1,2,3,4,5)) +
  geom_ribbon(aes(ymin = lower_ci, ymax = upper_ci, by = event, fill = event), alpha = 0.20) +
  theme_apa() +
  labs(x = "Deviation from mean age", y = "Challenge") +
  theme(legend.position = "none", 
        legend.text = element_text(size = 14), 
        axis.title = element_text(size = 14), 
        axis.text = element_text(size = 14), 
        legend.title = element_text(size = 14))

## Change in world views
res_changeww_vacation <- predict_gam(gam_changeww_vacation, length_out = 1000) 
res_changeww_vacation$event <- "Vacation"
res_changeww_occasion <- predict_gam(gam_changeww_occasion, length_out = 1000)
res_changeww_occasion$event <- "Special occasion"
res_changeww_illness <- predict_gam(gam_changeww_illness, length_out = 1000)
res_changeww_illness$event <- "Illness or injury"
res_changeww_death<- predict_gam(gam_changeww_death, length_out = 1000)
res_changeww_death$event <- "Death of a loved one"

res_changeww_graph <- rbind(res_changeww_vacation, 
                         res_changeww_occasion, 
                         res_changeww_illness, 
                         res_changeww_death)

plot_changeww_normativity <- ggplot(aes(x = normativity, y = ecq.changeww), data = res_changeww_graph) +
  geom_line(aes(by = event, colour = event), size = 1.3) +
  scale_y_continuous(limits = c(0,6)) +
    scale_color_manual(values = c(1,2,3,4,5)) +
  scale_fill_manual(values = c(1,2,3,4,5)) +
  geom_ribbon(aes(ymin = lower_ci, ymax = upper_ci, by = event, fill = event), alpha = 0.20) +
  theme_apa() +
  labs(x = "Deviation from mean age", y = "Change in world views") +
  theme(legend.position = "none", 
    legend.text = element_text(size = 14), 
        axis.title = element_text(size = 14), 
        axis.text = element_text(size = 14), 
        legend.title = element_text(size = 14))

## Emotional significance
res_emosig_vacation <- predict_gam(gam_emosig_vacation, length_out = 1000) 
res_emosig_vacation$event <- "Vacation"
res_emosig_occasion <- predict_gam(gam_emosig_occasion, length_out = 1000)
res_emosig_occasion$event <- "Special occasion"
res_emosig_illness <- predict_gam(gam_emosig_illness, length_out = 1000)
res_emosig_illness$event <- "Illness or injury"
res_emosig_death<- predict_gam(gam_emosig_death, length_out = 1000)
res_emosig_death$event <- "Death of a loved one"

res_emosig_graph <- rbind(res_emosig_vacation, 
                         res_emosig_occasion, 
                         res_emosig_illness, 
                         res_emosig_death)

plot_emosig_normativity <- ggplot(aes(x = normativity, y = ecq.emosig), data = res_emosig_graph) +
  geom_line(aes(by = event, colour = event), size = 1.3) +
  scale_y_continuous(limits = c(0,6)) +
    scale_color_manual(values = c(1,2,3,4,5)) +
  scale_fill_manual(values = c(1,2,3,4,5)) +
  geom_ribbon(aes(ymin = lower_ci, ymax = upper_ci, by = event, fill = event), alpha = 0.20) +
  theme_apa() +
  labs(x = "Deviation from mean age", y = "Emotional significance") +
  theme(legend.position = "none", 
    legend.text = element_text(size = 14), 
        axis.title = element_text(size = 14), 
        axis.text = element_text(size = 14), 
        legend.title = element_text(size = 14))

## External control
res_extcon_vacation <- predict_gam(gam_extcon_vacation, length_out = 1000) 
res_extcon_vacation$event <- "Vacation"
res_extcon_occasion <- predict_gam(gam_extcon_occasion, length_out = 1000)
res_extcon_occasion$event <- "Special occasion"
res_extcon_illness <- predict_gam(gam_extcon_illness, length_out = 1000)
res_extcon_illness$event <- "Illness or injury"
res_extcon_death<- predict_gam(gam_extcon_death, length_out = 1000)
res_extcon_death$event <- "Death of a loved one"

res_extcon_graph <- rbind(res_extcon_vacation, 
                         res_extcon_occasion, 
                         res_extcon_illness, 
                         res_extcon_death)

plot_extcon_normativity <- ggplot(aes(x = normativity, y = ecq.extcon), data = res_extcon_graph) +
  geom_line(aes(by = event, colour = event), size = 1.3) +
  scale_y_continuous(limits = c(0,6)) +
    scale_color_manual(values = c(1,2,3,4,5)) +
  scale_fill_manual(values = c(1,2,3,4,5)) +
  geom_ribbon(aes(ymin = lower_ci, ymax = upper_ci, by = event, fill = event), alpha = 0.20) +
  theme_apa() +
  labs(x = "Deviation from mean age", y = "External control") +
  theme(legend.position = "none", 
    legend.text = element_text(size = 14), 
        axis.title = element_text(size = 14), 
        axis.text = element_text(size = 14), 
        legend.title = element_text(size = 14))

## Extraordinariness
res_extrao_vacation <- predict_gam(gam_extrao_vacation, length_out = 1000) 
res_extrao_vacation$event <- "Vacation"
res_extrao_occasion <- predict_gam(gam_extrao_occasion, length_out = 1000)
res_extrao_occasion$event <- "Special occasion"
res_extrao_illness <- predict_gam(gam_extrao_illness, length_out = 1000)
res_extrao_illness$event <- "Illness or injury"
res_extrao_death<- predict_gam(gam_extrao_death, length_out = 1000)
res_extrao_death$event <- "Death of a loved one"

res_extrao_graph <- rbind(res_extrao_vacation, 
                         res_extrao_occasion, 
                         res_extrao_illness, 
                         res_extrao_death)

plot_extrao_normativity <- ggplot(aes(x = normativity, y = ecq.extrao), data = res_extrao_graph) +
  geom_line(aes(by = event, colour = event), size = 1.3) +
  scale_y_continuous(limits = c(0,6)) +
    scale_color_manual(values = c(1,2,3,4,5)) +
  scale_fill_manual(values = c(1,2,3,4,5)) +
  geom_ribbon(aes(ymin = lower_ci, ymax = upper_ci, by = event, fill = event), alpha = 0.20) +
  theme_apa() +
  labs(x = "Deviation from mean age", y = "Extraordinariness") +
  theme(legend.position = "none", 
    legend.text = element_text(size = 14), 
        axis.title = element_text(size = 14), 
        axis.text = element_text(size = 14), 
        legend.title = element_text(size = 14))

## Impact
res_impact_vacation <- predict_gam(gam_impact_vacation, length_out = 1000) 
res_impact_vacation$event <- "Vacation"
res_impact_occasion <- predict_gam(gam_impact_occasion, length_out = 1000)
res_impact_occasion$event <- "Special occasion"
res_impact_illness <- predict_gam(gam_impact_illness, length_out = 1000)
res_impact_illness$event <- "Illness or injury"
res_impact_death<- predict_gam(gam_impact_death, length_out = 1000)
res_impact_death$event <- "Death of a loved one"

res_impact_graph <- rbind(res_impact_vacation, 
                         res_impact_occasion, 
                         res_impact_illness, 
                         res_impact_death)

plot_impact_normativity <- ggplot(aes(x = normativity, y = ecq.impact), data = res_impact_graph) +
  geom_line(aes(by = event, colour = event), size = 1.3) +
  scale_y_continuous(limits = c(0,6)) +
    scale_color_manual(values = c(1,2,3,4,5)) +
  scale_fill_manual(values = c(1,2,3,4,5)) +
  geom_ribbon(aes(ymin = lower_ci, ymax = upper_ci, by = event, fill = event), alpha = 0.20) +
  theme_apa() +
  labs(x = "Deviation from mean age", y = "Impact") +
  theme(legend.position = "none", 
    legend.text = element_text(size = 14), 
        axis.title = element_text(size = 14), 
        axis.text = element_text(size = 14), 
        legend.title = element_text(size = 14))

## Predictability
res_predict_vacation <- predict_gam(gam_predict_vacation, length_out = 1000) 
res_predict_vacation$event <- "Vacation"
res_predict_occasion <- predict_gam(gam_predict_occasion, length_out = 1000)
res_predict_occasion$event <- "Special occasion"
res_predict_illness <- predict_gam(gam_predict_illness, length_out = 1000)
res_predict_illness$event <- "Illness or injury"
res_predict_death<- predict_gam(gam_predict_death, length_out = 1000)
res_predict_death$event <- "Death of a loved one"

res_predict_graph <- rbind(res_predict_vacation, 
                         res_predict_occasion, 
                         res_predict_illness, 
                         res_predict_death)

plot_predict_normativity <- ggplot(aes(x = normativity, y = ecq.predict), data = res_predict_graph) +
  geom_line(aes(by = event, colour = event), size = 1.3) +
  scale_y_continuous(limits = c(0,6)) +
    scale_color_manual(values = c(1,2,3,4,5)) +
  scale_fill_manual(values = c(1,2,3,4,5)) +
  geom_ribbon(aes(ymin = lower_ci, ymax = upper_ci, by = event, fill = event), alpha = 0.20) +
  theme_apa() +
  labs(x = "Deviation from mean age", y = "Predictability") +
  theme(legend.position = "none", 
    legend.text = element_text(size = 14), 
        axis.title = element_text(size = 14), 
        axis.text = element_text(size = 14), 
        legend.title = element_text(size = 14))

## Social status change
res_socstat_vacation <- predict_gam(gam_socstat_vacation, length_out = 1000) 
res_socstat_vacation$event <- "Vacation"
res_socstat_occasion <- predict_gam(gam_socstat_occasion, length_out = 1000)
res_socstat_occasion$event <- "Special occasion"
res_socstat_illness <- predict_gam(gam_socstat_illness, length_out = 1000)
res_socstat_illness$event <- "Illness or injury"
res_socstat_death<- predict_gam(gam_socstat_death, length_out = 1000)
res_socstat_death$event <- "Death of a loved one"

res_socstat_graph <- rbind(res_socstat_vacation, 
                         res_socstat_occasion, 
                         res_socstat_illness, 
                         res_socstat_death)

plot_socstat_normativity <- ggplot(aes(x = normativity, y = ecq.socstat), data = res_socstat_graph) +
  geom_line(aes(by = event, colour = event), size = 1.3) +
  scale_y_continuous(limits = c(0,6)) +
    scale_color_manual(values = c(1,2,3,4,5)) +
  scale_fill_manual(values = c(1,2,3,4,5)) +
  geom_ribbon(aes(ymin = lower_ci, ymax = upper_ci, by = event, fill = event), alpha = 0.20) +
  theme_apa() +
  labs(x = "Deviation from mean age", y = "Social status change") +
  theme(legend.position = "none", 
    legend.text = element_text(size = 14), 
        axis.title = element_text(size = 14), 
        axis.text = element_text(size = 14), 
        legend.title = element_text(size = 14))

## Valence
res_val_vacation <- predict_gam(gam_val_vacation, length_out = 1000) 
res_val_vacation$event <- "Vacation"
res_val_occasion <- predict_gam(gam_val_occasion, length_out = 1000)
res_val_occasion$event <- "Special occasion"
res_val_illness <- predict_gam(gam_val_illness, length_out = 1000)
res_val_illness$event <- "Illness or injury"
res_val_death<- predict_gam(gam_val_death, length_out = 1000)
res_val_death$event <- "Death of a loved one"

res_val_graph <- rbind(res_val_vacation, 
                         res_val_occasion, 
                         res_val_illness, 
                         res_val_death)

plot_val_normativity <- ggplot(aes(x = normativity, y = ecq.val), data = res_val_graph) +
  geom_line(aes(by = event, colour = event), size = 1.3) +
  scale_y_continuous(limits = c(0,6)) +
    scale_color_manual(values = c(1,2,3,4,5)) +
  scale_fill_manual(values = c(1,2,3,4,5)) +
  geom_ribbon(aes(ymin = lower_ci, ymax = upper_ci, by = event, fill = event), alpha = 0.2, fullrange = TRUE) +
  theme_apa() +
  labs(x = "Deviation from mean age", y = "Valence", color = "Event", fill = "Event") +
  theme(legend.position = "none", 
    legend.text = element_text(size = 14), 
        axis.title = element_text(size = 14), 
        axis.text = element_text(size = 14), 
        legend.title = element_text(size = 14))

plot_val_normativity2 <- ggplot(aes(x = normativity, y = ecq.val), data = res_val_graph) +
  geom_line(aes(by = event, colour = event), size = 1.3) +
  scale_y_continuous(limits = c(0,6)) +
    scale_color_manual(values = c(1,2,3,4,5)) +
  scale_fill_manual(values = c(1,2,3,4,5)) +
  geom_ribbon(aes(ymin = lower_ci, ymax = upper_ci, by = event, fill = event), alpha = 0.2, fullrange = TRUE) +
  theme_apa() +
  labs(x = "Deviation from mean age", y = "Valence", color = "Event", fill = "Event") +
  theme(legend.position = "right", 
    legend.text = element_text(size = 14), 
        axis.title = element_text(size = 14), 
        axis.text = element_text(size = 14), 
        legend.title = element_text(size = 14))

legend <- cowplot::get_legend(plot_val_normativity2)

cowplot::plot_grid(plot_chall_normativity, plot_changeww_normativity, plot_emosig_normativity, 
                   plot_extcon_normativity, plot_extrao_normativity, plot_impact_normativity, 
                   plot_predict_normativity, plot_socstat_normativity, plot_val_normativity, 
                   NULL, legend, NULL, ncol = 3, nrow = 4, 
                   rel_heights = c(1,1,1,0.5))
```

```
## ggsave("Plot5.svg", height = 13, width = 10)
```
